# Lipid Extract from *Ulva ohnoi* Algae: Chemical Characterization, Antibacterial Potential, and Cytotoxicity in the Search for New Agents Against MDR Infections

**DOI:** 10.1007/s10126-026-10717-z

**Published:** 2026-10-17

**Authors:** Marisa Castro Jara, Diego Serrasol do Amaral, Leonardo Werner, Eduardo Bastos, Luana de Azevedo Aimi, Nathalia Stark Pedra, Vithor Parada Garcia, Matheus Pereira de Albuquerque, Clarissa Piccinin Frizzo, Milena de Nardin, Wellington da Rocha da Silva, Silvia de Oliveira Hübner, Rodrigo de Almeida Vaucher, Cláudio Martins Pereira de Pereira, Patrícia da Silva Nascente

**Affiliations:** 1 Department of Microbiology and Parasitology, Institute of Biology, Federal University of Pelotas, Pelotas, RS Brazil; 2 Department of Biochemistry and Bioprospecting, Federal University of Pelotas, Pelotas, RS Brazil; 3 Department of Preventive Veterinary Medicine, Faculty of Veterinary Medicine, Federal University of Pelotas, Pelotas, RS Brazil; 4 Department of Botany, Federal University of Santa Catarina, Florianópolis, SC Brazil; 5 Laboratory of Research in Biochemistry and Molecular Biology of Microorganisms, Federal University of Pelotas, Pelotas, RS Brazil; 6 Laboratory of Neurochemistry, Inflammation, and Cancer, Federal University of Pelotas, Pelotas, RS Brazil; 7 Laboratory of Ionic Liquids and Nanomaterials, Department of Chemistry, Federal University of Santa Maria, Santa Maria, RS Brazil

**Keywords:** Antibiotics, Seaweed, Chlorophyta, Green macroalgae, Green tide, Sea lettuce

## Abstract

Antimicrobial resistance is a critical global concern. This study explored the lipid extract (LE) of *Ulva ohnoi* regarding its chemical composition, antibacterial activity, and cytotoxicity against multidrug-resistant (MDR) microorganisms. The LE was characterized by GC–MS, thermogravimetric analysis (TGA), and differential scanning calorimetry (DSC). Antibacterial efficacy was determined by Minimum Inhibitory Concentration (MIC) and Minimum Bactericidal Concentration (MBC) against MDR isolates: *Acinetobacter baumannii*, *Escherichia coli*, *Klebsiella pneumoniae*, *Pseudomonas aeruginosa*, *Staphylococcus aureus*, and coagulase-negative Staphylococci (CoNS). Cytotoxicity was evaluated in murine fibroblast (L929) and bovine renal fibroblast (MDBK) cells using MTT, Neutral Red (NR), Lactate Dehydrogenase (LDH), and hemolytic activity (HA) assays. Lipid characterization revealed palmitic acid (63.62%) and oleic acid (21.01%) as major components. TGA indicated 88% mass loss, and DSC showed no amorphous/crystalline domains. LE showed favorable MIC values against *S. aureus* and CoNS (1.33 mg/mL) and against *A. baumannii* and *E. coli* (2 mg/mL), indicating moderate antibacterial activity typical of an unfractionated crude extract. MBC results indicated bacteriostatic activity (1–8 mg/mL). The extract was cytotoxic to L929 and MDBK cells at concentrations close to its MIC (IC₅₀ 0.25–1.95 mg/mL) but well tolerated by blood cells (HA IC_50_ 4.705 mg/mL). *U. ohnoi* LE extends, rather than establishes, the previously reported antibacterial potential of *Ulva* lipids through comprehensive chemical characterization and MDR-isolate evaluation. However, low selectivity indices (SI < 10) indicate that the LE does not yet present an adequate therapeutic window, reinforcing the need for bioassay-guided purification. Nevertheless, it is a starting point for developing new antibacterial agents.

**Graphical Abstract:**

**Figure:**
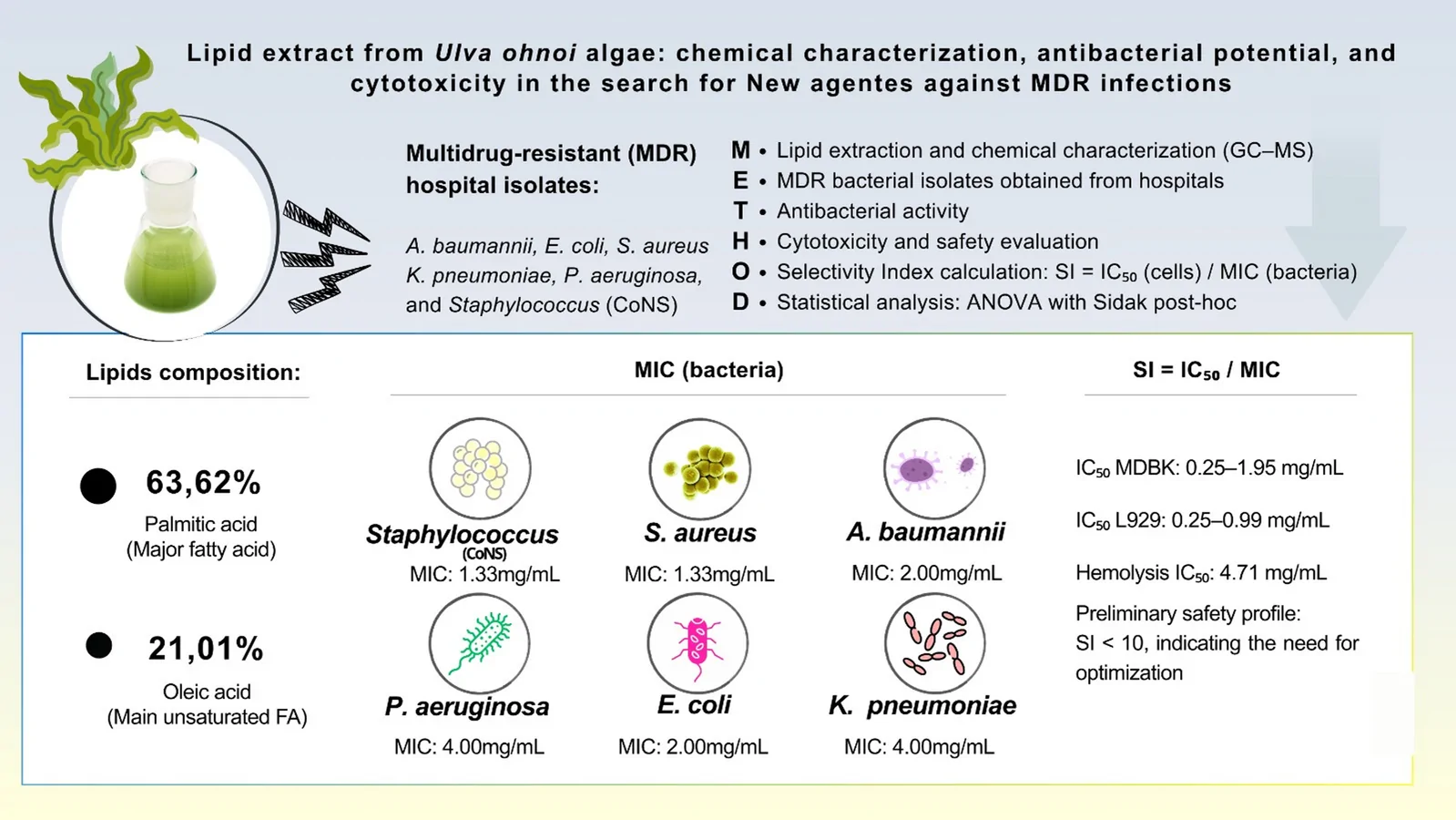

**Supplementary Information:**

The online version contains supplementary material available at https://doi.org/10.1007/s10126-026-10717-z.

## Introduction

*Ulva* algae have a rich source of long-chain PUFAs, especially C18 PUFAs such as α-linolenic acid (C18:3 n-3), stearidonic acid (C18:4 n-3), and linoleic acid (C18:2 n-6) (Kumari et al. 2013; Moreira et al. 2021). Species of the genus *Ulva*, commonly known as “sea lettuce,” belong to the Chlorophyta group and are particularly rich in carbohydrates, including sulfated heteropolysaccharides (Hmani et al. 2024; Rodríguez-Iglesias et al. 2024). In addition, they have a diverse composition, containing proteins, vitamins, minerals, amino acids, and PUFAs (Rocha et al. 2023).

Algae of the genus *Ulva* exhibit antibacterial, anti-inflammatory (Kang et al. 2021; Kidgell et al. 2020), antitumor (García-Márquez et al. 2023; Wahlström et al. 2020), anticoagulant, antioxidant (Don et al. 2021; Wahlström et al. 2020), immunomodulatory (Montuori et al. 2022; Pratt et al. 1944), and antihyperlipidemic (Don et al. 2021; Yuan et al. 2018) activities. These properties have led to applications for *Ulva* species, including pharmaceutical products, food, animal feed, and bioremediation (Don et al. 2021; Simon et al. 2024; Tran et al. 2023). Also, *Ulva* biomass can be used as a raw material for biodegradable polymers for packaging (Guidara et al. 2020; Manikandan and Lens 2023) and biofuel production (Biancarosa et al. 2018; Rodríguez-Iglesias et al. 2024; Simon et al. 2022).

MDR has become a global concern due to the alarming increase in morbidity and mortality rates (Bilal et al. 2024; Laitin et al. 2024; Oliveira et al. 2024). The World Health Organization (WHO) considers multidrug-resistant (MDR) bacteria one of the ten greatest threats to global public health (Oliveira et al. 2024; World Health Organization 2023). Addressing this problem requires urgent and coordinated actions that integrate different sectors, aiming to achieve the health-related targets of the Sustainable Development Goals (SDGs). One of the 17 targets is the goal of ending, by 2030, epidemics such as AIDS, tuberculosis, malaria, and neglected tropical diseases, while strengthening measures to control hepatitis, waterborne diseases, and other communicable diseases (Ferdinand et al. 2023; ONU Brasil 2015; Organização Pan-Americana de Saúde 2020).

In parallel, the effectiveness of currently available ATBs has been compromised by the emergence of MDR bacteria (Alreshidi et al. 2023; Camou et al. 2017; Talbot et al. 2019). Rifampicin resistance, for example, was identified in 2017 as an important concern because of its use in the treatment of tuberculosis that affects approximately 10 million people annually and is associated with 1.5 million deaths worldwide (Organização Pan-Americana de Saúde 2020).

Given the urgent need for new antibacterial agents and the versatility of algae, attention has been directed towards it. Their ability to adapt to different environments and their potential for large-scale biomass production make them particularly attractive for this purpose, especially during periods of massive *Ulva* spp. proliferation, known as green tides, which have become more frequent and widespread in recent decades (Ren et al. 2024; Zeng et al. 2024; Wahlström et al. 2020; Wang et al. 2022).

Despite growing interest in the genus *Ulva* as a source of bioactive compounds, the vast majority of published studies on *U. ohnoi* have focused on its sulfated polysaccharide fraction (ulvan), evaluated for antioxidant activity (Glasson et al. 2017; Kidgell et al. 2020), immunomodulatory properties in aquaculture models (Ponce et al. 2020), and structural characterization. To date, only one study has examined the biological activity of the lipid fraction of *U. ohnoi*: Kang et al. (2021) demonstrated antibacterial activity of *U. ohnoi* oil against non-resistant reference strains using a qualitative agar-based diffusion method at a fixed concentration, and anti-inflammatory effects in an animal model of colitis, a valuable contribution that, by its own scope, focused on the endpoints and did not extend to quantitative Minimum Inhibitory Concentration (MIC) and Minimum Bactericidal Concentration (MBC) determination, testing against clinically relevant MDR pathogens, or cytotoxicity assessment in mammalian cell lines.

These aspects therefore remain to be explored. Accordingly, the lipid fraction of *U. ohnoi* remains largely unexplored in the context of MDR pathogens and pharmacological safety, a significant gap given that fatty acid (FA)-rich lipid extracts (LE) from green macroalgae are recognized sources of membrane-active antimicrobial compounds with mechanisms of action distinct from conventional antibiotics, and therefore potentially capable of circumventing established resistance mechanisms (Desbois and Smith 2010). The present study addresses this gap by providing, to our knowledge, the first evaluation of *U. ohnoi* LE against hospital-acquired MDR isolates using standardized MIC and MBCmethodology (Clinical and Laboratory Standards Institute (CLSI) 2024), combined with Gas Chromatography Coupled with Mass Spectrometry (GC–MS) characterization of the FA profile, thermal analysis by thermogravimetric analysis (TGA), and differential scanning calorimetry (DSC), and a multidimensional cytotoxicity assessment using four independent assays (3-(4,5-dimethylthiazol-2-yl)−2,5-diphenyltetrazolium bromide) (MTT), Neutral Red (NR), Lactate Dehydrogenase (LDH), and hemolytic activity (HA), across two mammalian cell lines, a combination not previously reported for any lipid fraction of this species. To date, only one study has investigated the biological activity of the lipid fraction of *U. ohnoi*. Kang et al. (2021) demonstrated the antibacterial activity of *U. ohnoi* oil against non-resistant reference strains using a qualitative agar-based diffusion method at a fixed concentration, as well as anti-inflammatory effects in an animal model of colitis. Although these findings represent a valuable contribution, the scope of that study did not include quantitative determination of MIC and MBC, evaluation against clinically relevant MDR pathogens, or cytotoxicity assessment in mammalian cell lines. These aspects therefore remain largely unexplored.

Therefore, investigating the antibacterial potential of *U. ohnoi* LE, together with its chemical composition, thermal stability, and cytotoxicity in mammalian cell models, particularly against multidrug-resistant hospital isolates, is essential. Such information may provide a basis for future investigations into the pharmaceutical potential of this abundant marine and freshwater biomass as a source of novel antibacterial compounds.

## Materials and Methods

### Collection of the Macroalga *Ulva ohnoi*

The algal biomass *U. ohnoi* was collected from an aquaculture facility located at 27° 35′ 4.64″ S, 48° 26′ 31.10″ W in Florianópolis-SC, Brazil. The samples were promptly transported to the Laboratory of Phycology at the Federal University of Santa Catarina (LAFIC–UFSC) within one hour, using light-protected thermal containers. Upon arrival, the biomass was rinsed with UV-C sterilized seawater, carefully brushed to remove epiphytes, invertebrates, and sediments, then dried in an oven at 60 °C to a constant weight and frozen at −80 °C until further analysis. Species-level identification was performed according to Salvi et al. (2021).

### Lipid Extraction Procedure

A 10 g aliquot of dried *U. ohnoi* biomass was ground in a mortar and pestle. Lipids were extracted according to the method described by Bligh and Dyer (1959). 60 mL of a chloroform–methanol solution in a 1:2 ratio was added to the sample and the mixture was homogenized in sealed flasks for 1 h. After this step, the mixture was centrifuged for 20 min at 3000 rpm. The aqueous phase was discarded, and the solvent from the lipid phase was removed using a rotary evaporator, leaving the LE. The extract was resuspended in hexane, and the solvent was again removed using a rotary evaporator to remove impurities. The resulting LE was stored in light-protected flasks at − 20 °C until further analysis.

### Gas Chromatography Coupled to Mass Spectrometry (GC–MS)

The LE of *U. ohnoi* was subjected to derivatization with boron trifluoride (BF₃). Briefly, a 250-mg aliquot of the LE was transferred to a glass flask, and 6 mL of a 2% (w/v) methanolic sodium hydroxide solution was added. The mixture was refluxed with stirring for 5 min. Subsequently, 5 mL of BF₃/methanol (MeOH) was added, and the mixture was refluxed for an additional 5 min. The solution was then transferred to a separatory funnel, and 20 mL of hexane was added to promote phase separation. After discarding the aqueous phase, the organic phase was filtered through sodium sulfate into a glass flask. The solvent was evaporated using a rotary evaporator, and the residue was eluted in 1 mL of HPLC-grade hexane (≥ 99.9%) and transferred to vials for GC–MS analysis. To determine the FA composition of the *U. ohnoi* LE, a Shimadzu gas chromatograph (GC-2010) coupled to a mass spectrometer (GCMS-QP2020) and equipped with an SP-2560 fused-silica capillary column (100 m × 0.25 mm × 0.10 μm) was used. The injection volume was 8 μL in split mode at a split ratio of 1:50. The interface and ion source temperatures were set at 280 °C and 200 °C, respectively, while the injector temperature was maintained at 200 °C. Helium was used as the carrier gas at a flow rate of 3 mL min⁻^1^. The initial column temperature was 100 °C and was maintained for 5 min, followed by heating at a rate of 5 °C min⁻^1^ to 250 °C, resulting in a total analysis time of 55 min. The mass spectrometer was operated in scan mode over a detection range of 35–700 m/z, allowing identification of the compounds. Lipid extraction and GC–MS analyses were performed at the Laboratory of Innovation and Solutions in Chemistry (INNOVASCHEM), Federal University of Pelotas (UFPel).

### Thermogravimetric Analyses (TGA)

TGA of *U. ohnoi* were performed using a TA Instruments Q5000 thermogravimetric analyzer (USA). To ensure measurement accuracy, the instrument was previously calibrated with 99.9% pure calcium oxalate monohydrate (CaC₂O₄ · H₂O). Thermal characterization was performed at a constant heating rate of 10 °C min⁻^1^ up to 650 °C. Each analysis used approximately 3 mg of *U. ohnoi*. The measurements were conducted under an inert nitrogen (N₂) atmosphere at a flow rate of 25 mL min⁻^1^. No sample drying or isothermal procedure was performed before the beginning of the analysis.

### Differential Scanning Calorimetry (DSC)

The thermal behavior of *U. ohnoi* was characterized by DSC to investigate its thermal properties, which are directly related to phase transitions and their respective enthalpy changes. Phase transitions were specifically determined using Modulated Temperature Differential Scanning Calorimetry (MTDSC), with Q2000 DSC instrument (TA Instruments, USA). The mass of the *U. ohnoi* LE samples ranged from 1 to 5 mg, and samples weighed with an accuracy of ± 0.001 mg on a Sartorius M500P analytical balance. All samples were subjected to a total of three heating and cooling cycles. Measurements were conducted over a temperature range extending from −80 °C to 300 °C. The heating rate used was 10 °C min⁻^1^, and nitrogen (N₂) was used as the inert purge gas at a flow rate of 50 mL min⁻^1^.

### Multidrug-resistant (MDR) Bacterial Isolates

The study included Gram-negative bacilli (GNB) and Gram-positive cocci (GPC), with three bacterial isolates from each of the following species: *A. baumannii*, *E. coli*, *K. pneumoniae*, and *P. aeruginosa*, *S. aureus*, and CoNS. The bacterial isolates were provided by two hospitals in the city of Pelotas-RS, Brazil. The resistance profile of the isolates was analyzed using automated VITEK 2-BioMérieux and BD PHOENIX and was characterized by resistance to more than three classes of ATB to be considered MDR. The isolates were stored in the Bacteriotheque of the Microbiology and Bioprospecting Laboratory of the Department of Microbiology and Parasitology (DEMP) of the Institute of Biology at UFPel and were previously approved in the earlier study by Jara et al. (2021) by the Research Ethics Committee (CEP) under numbers 2,961,379 and 2,985,372, as well as by the National Research Ethics Commission (CONEP) under number 2,880,831, registered on the Brazil Platform.

#### Minimum Inhibitory Concentration (MIC)

The antimicrobial activity of *U. ohnoi* LE was evaluated using the MIC method. This endpoint was selected as the primary screening approach for the initial antimicrobial characterization of *U. ohnoi* LE, following an adaptation of the Clinical and Laboratory Standards Institute (CLSI, 2024) M07-A12 manual for a natural product. Time-kill kinetic studies are planned for subsequent investigations. For this purpose, a 16 mg/mL solution was prepared by dissolving 100 mg of algal LE in 2 mL of absolute ethanol, followed by the addition of 5.257 mL of Mueller–Hinton broth (MHB). In sterile 96-well microdilution plates containing 100 μL of MHB, 100 μL of the *U. ohnoi* LE solution (16 mg/mL) was added to the second column. Subsequently, ten serial dilutions, ranging from 8.000 to 0.015 mg/mL, were performed from the second to the eleventh column, with a final volume of 100 μL per well. The MDR bacterial isolates were cultured on Brain Heart Infusion (BHI) agar plates overnight at 37 °C for 24 h before the assay. An inoculum was prepared by suspending a portion of the bacterial colony in 3 mL of saline solution until a turbidity equivalent to 0.5 on the McFarland scale (1.5 × 10^8^ CFU/mL) was reached. A 0.5-mL aliquot of this suspension was then transferred to 4.5 mL of MHB, resulting in a bacterial suspension of 1.5 × 10^7^ CFU/mL. The first column served as the negative control and contained only MHB, whereas the last column served as the positive growth control and contained MHB plus the bacterial inoculum. Subsequently, 5 μL of the inoculum was added to the wells from the second to the twelfth columns. The plates were incubated at 37 °C for 24 h. The MIC was subsequently determined using a colorimetric method by adding 40 μL/well of 0.015% 2,3,5-triphenyltetrazolium chloride (TTC), followed by incubation at 37 °C for 30 min. The results were determined visually based on the presence or absence of coloration. The assay for each isolate was performed in duplicate, with three independent repetitions conducted on different dates. Readings were performed by a single trained analyst. Reproducibility was ensured through duplicate wells and three independent repetitions on separate dates, with discrepant replicates repeated before assigning the final MIC value. No external antibiotic reference compound was included as a positive control because the isolates had been previously characterized as resistant to standard antibiotics by automated susceptibility testing (VITEK 2/BD PHOENIX; Supplementary Table 1). Assay validity was instead ensured using internal negative (broth only) and positive (broth + inoculum) growth controls on each plate.

#### Minimum Bactericidal Concentration (MBC)

The brain–heart agar (BHA) plating method was used to evaluate the MBC immediately after the MIC assay. A Mueller–Hinton agar (MHA) plate was inoculated with a 5 μL aliquot from each well in which the LE of *U. ohnoi* showed antibacterial activity as well as from the subsequent well in which no antibacterial activity was observed. The plates were then incubated at 37 °C for 24 h. Colony growth was assessed visually, and the results were classified as bacteriostatic (inhibition of bacterial growth, with colony formation) or bactericidal (bacterial death, with no colony formation). The assays for each isolate were performed in duplicate and with three repetitions on different dates. When bacterial growth was observed in the subculture at all tested concentrations—including the highest concentration in the dilution series (8 mg/mL)—the isolate was classified as bacteriostatic, and the MBC was recorded as greater than the highest concentration tested (MBC/MIC > 4), according to the criteria described by Akinwale et al. (2024).

### Cell Viability Assessment

#### Hemolytic Activity (HA)

HA assay was performed according to Vaucher et al. (2010), using defibrinated sheep red blood cells. The red blood cells were washed and resuspended three times in phosphate-buffered saline (PBS) to obtain a final concentration of 4% (v/v). 8 mg/mL LE of *U. ohnoi* diluted in 1 mL of the 4% red blood cell suspension was added, and serial dilutions were performed up to a concentration of 0.0625 mg/mL. The samples were incubated for 2 h at 37 °C with agitation in a shaker incubator and then centrifuged for 10 min at 2000 rpm, and the supernatant was transferred to a 96-well plate. The negative control consisted of a 2.3% EtOH solution in water, and the positive control was TritonX-100. Hemoglobin release was read using a Hitachi U-1100 spectrophotometer (Hitachi), at 425 to 450 nm. The percentage of hemolysis was calculated using the following equation:

$$\left[\left(\mathrm{Abs}\;\mathrm{sample}-\mathrm{Abs}\;\mathrm{blank}\right)/\left(\mathrm{Abs}\;\mathrm{ctrl}-\mathrm{Abs}\;\mathrm{blank}\right)\times100\right]$$

Where: Abs sample was the absorbance of hemoglobin release in the supernatant, Abs blank was the absorbance of the negative control, and Abs ctrl was the absorbance of the positive control. The analyses were performed in triplicate. A color control assay of *U. ohnoi* LE was performed to observe the interference of the extract color in serial dilutions at the same concentrations performed in duplicate in the HA test.

#### Cell Culture

Cell viability was assessed by Madin-Darby-Bovine-Kidney (MDBK) and mouse fibroblast (L929) cells obtained from Laboratory of Virology and Immunology at UFPel. The cells were propagated in Modified Eagle Medium (MEM) (pH 7.4) enriched with penicillin/streptomycin (100 U/L) and amphotericin B (0.1%), together with 10% (v/v) fetal bovine serum (FBS). Cells were seeded at 100 μL per well in 96-well plates and maintained under standard culture conditions (37 °C in a humidified incubator containing 5% CO₂) until confluence was reached (24 h).

#### Cell Treatment

*U. ohnoi* LE was dissolved in 15% (v/v) ethanol (EtOH) in water to prepare a stock solution of 52 mg/mL. This stock solution was subsequently diluted in MEM to obtain an initial concentration of 8 mg/mL with 2.3% (v/v) EtOH in MEM. In a 96-well plate, 100 μL of MEM without FBS was added in all wells of rows A, B, C, and D (quadruplicate), except for column 1, in which 200 μL of the 8 mg/mL LE solution was added in the first well of column 1. Serial 1:2 dilutions were then performed by transferring 100 μL of the solution from the first column to the adjacent well containing 100 μL of MEM. The final LE concentrations were 8, 4, 2, 1, 0.5, 0.25, 0.125, 0.0625, 0.03125, 0.015625, and 0.0078125 mg/mL. The EtOH concentration decreased proportionally with each dilution from 2.3% in the first well to 0.00225% in the eleventh well. A MEM control was included in the final column. For the cell viability assay, 30,000 cells/well were treated with these concentrations for 24 h, and then the MTT assay was performed. The assays were conducted in quadruplicate.

#### Cell Viability–MTT

Quantitative assessment of cell viability was performed using the MTT assay, according to the protocol described by Mosmann (1983). This colorimetric technique is based on the ability of functional mitochondria in viable cells to reduce MTT to blue formazan crystals. Briefly, the wells were washed with calcium- and magnesium-free balanced saline solution (CMF; pH 7.4) and exposed to a 0.5 mg/mL MTT solution, followed by a 4-h incubation under standard culture conditions. The resulting formazan crystals were solubilized in 50 μL of absolute EtOH, and the absorbance was measured at 570 nm using a microplate reader (Biotex ELX800). Concentrations that did not show cytotoxicity were considered capable of preserving cell viability close to 100% relative to the control (untreated cells). The assays were conducted in quadruplicate. The results were expressed as a percentage relative to the control using the following equation:

$$\mathrm{Cell}\;\mathrm{viability}(\%)=({\mathrm{OD}}_{570}\;\mathrm{of}\;\mathrm{treated}\;\mathrm{cells}/{\mathrm{OD}}_{570}\;\mathrm{of}\;\mathrm{control})\times\;100$$

#### Cell Viability EtOH Test – MTT

To assess the possible interference of EtOH on cell viability, a parallel assay was performed exposing the MDBK and L929 strains to decreasing concentrations of EtOH p.a. in MEM. The concentrations tested (2.3; 1.15; 0.575; 0.287; 0.143; 0.071; 0.035; 0.017; 0.008; 0.004 and 0.002%) corresponded exactly to those present in the *U. ohnoi* LE dilutions. The final column contained a negative control consisting of MEM without EtOH.

This assay was conducted under the same conditions and simultaneously with the LE assay, using the MTT protocol described above to minimize inter-experimental variation. All assays were performed in quadruplicate. In addition to quantitative assays, cells were also monitored using light microscopy to observe morphological changes and cell integrity.

#### Cell Viability – Neutral Red (NR)

Cell viability was assessed using the NR assay, which is based on the uptake and accumulation of dye in the lysosomes of viable cells (Borenfreund and Puerner 1985). Following the cell treatment incubation period, the supernatant was removed and 200 μL of 0.033% NR solution was added to each of the 96 wells of the plate and incubated at 37 °C for 2 h in an incubator with 5% CO₂. The NR solution was then removed and the cells were washed with 100 μL of MEM without FBS, which was subsequently removed. The cells were then fixed and decolorized with 100 μL of 1% acetic acid/50% EtOH solution. The plates were shaken for 10 min and the absorbance was measured at 540 nm with a microplate reader, against a reference well containing no cells. Cell viability was calculated using the following equation:

$$\mathrm{Cell}\;\mathrm{viability}(\%)=({\mathrm{OD}}_{570}\;\mathrm{of}\;\mathrm{treated}\;\mathrm{cells}/{\mathrm{OD}}_{570}\;\mathrm{of}\;\mathrm{control})\times\;100$$

Concentrations that did not show cytotoxicity were considered to maintain cell viability close to 100% compared to the control (untreated cells). The assays were conducted in quadruplicate. In parallel with this quantitative assay, the cells were also monitored by light microscopy to observe morphological changes and cellular integrity.

### Lactate Dehydrogenase (LDH)

The LDH assay was performed by removing the supernatant from dilutions ranging from 0.5 to 0.007 mg/mL after 24 h of the cell treatment in the MTT assay to evaluate the plasma integrity of L929 and MDBK cells (Decker and Lohmann-Matthes 1988). LDH activity in the supernatant was determined using a LDH® reagent (Labtest, Diagnostic) in Cobas Miras-Roche® biochemical equipment. The assay is based on a kinetic reaction, in which LDH catalyzes the reduction of pyruvate with NADH, producing lactate and NAD +. The catalytic concentration is determined by the rate of NADH decomposition, measured by the decrease in absorbance at 340 nm. The analysis was performed in triplicate, and the values were determined as a percentage of LDH released.

### Calculation of the Selectivity Index (SI)

To evaluate the pharmacological safety of *U. ohnoi* LE, the selectivity index (SI) was calculated, to relate the cytotoxicity and antimicrobial activity of the extract. The SI corresponds to the ratio between the concentration that causes 50% inhibition of cell viability (IC₅₀) in eukaryotic cells and the MIC against the microorganisms tested, according to the equation:

$$SI=\frac{IC50}{MIC}$$

A selectivity index equal to or greater than 10 (SI ≥ 10) was considered an indicative parameter of a safe therapeutic margin, ensuring antimicrobial efficacy with minimal cell toxicity. The SI was calculated from the ratio between the IC₅₀ (obtained in the MTT, NR, and LDH viability assays in the MDBK and L929 cell lines) and the corresponding MIC values.

### Statistical Analysis

Data from the TGA and DSC analyses were processed using TA Universal Analysis 2000 software (version 4.5, TA Instruments, USA). MIC and MBC assays were performed in duplicate, with three technical replicates conducted on different dates, and the results were expressed as the mean ± standard deviation (SD) of six independent determinations (n = 6). LE treatment was performed in triplicate for the HA and LDH assays and in quadruplicate for the MTT and NR assays. The results were analyzed using two-way analysis of variance (ANOVA) with GraphPad Prism version 8.0 software (GraphPad Software, San Diego, USA). The results were expressed as mean ± standard error of the mean (SEM), and means were compared using Sidak's test. Differences were considered statistically significant at P < 0.05. It is important to note that, given the twofold dilution scale inherent to the CLSI M07-A12 (2024) microdilution method, standard deviations were calculated to reflect the discrete nature of the dilution series. Consequently, a standard deviation of zero may occur when replicate readings converge on the same dilution step, whereas non-zero, non-rounded values may occur when replicates fall into different dilution steps. This represents a genuine reflection of assay reproducibility.

## Results

### Lipid Extract Composition

The major FA composition of *U. ohnoi* is shown in Table 1. Gas chromatography analysis of FA methyl esters (FAMEs) revealed a significant predominance of palmitic acid, corresponding to 63.62% of the total relative area. Saturated fatty acids (SFAs) including myristic acid (C14:0; 1.24%), stearic acid (C18:0; 1.52%), and behenic acid (C22:0; 0.72%) were identified, resulting in a total SFA content of 67.10%. Monounsaturated fatty acids (MUFAs) accounted for 25.85%, consisting mainly of oleic acid (C18:1; 21.01%) and palmitoleic acid (C16:1; 4.84%). PUFAs accounted for 7.05%, including α-linolenic acid (C18:3; 3.93%), linoleic acid (C18:2; 1.77%), stearidonic acid (C18:4; 0.97%), and hexadecatetraenoic acid (C16:4; 0.37%).

**Table 1:** Fatty acid composition of the lipid extract from *Ulva ohnoi* analyzed by GC–MS, with identification of each fatty acid, chemical formula (C:n notation), and relative abundance (%)

Carbon chain:double bonds (C:n)	Relative area (%)	Fatty acid
C14:0	1.24	Myristic acid (methyl myristate)
C16:4	0.37	Hexadecatetraenoic acid (methyl ester)
C16:1	4.84	Palmitoleic acid (methyl palmitoleate)
C16:0	63.62	Palmitic acid (methyl palmitate)
C18:4	0.97	Stearidonic acid (methyl stearidonate)
C18:2	1.77	Linoleic acid (methyl linoleate)
C18:3	3.93	α-Linolenic acid (methyl α-linolenate)
C18:1	21.01	Oleic acid (methyl oleate)
C18:0	1.52	Stearic acid (methyl stearate)
C22:0	0.72	Behenic acid (methyl behenate)
**Σ SFA**	**67.10**	
**Σ MUFA**	**25.85**	
**Σ PUFA**	**7.05**	

Fatty acid composition of the *Ulva ohnoi* lipid extract analyzed by GC–MS, showing the identification of each fatty acid, carbon chain:double bonds (C:n notation), and relative abundance (%). Fatty acids are grouped by class: SFA,:saturated fatty acids; MUFA,:monounsaturated fatty acids; PUFA: polyunsaturated fatty acids. Relative area values ​​(%) were calculated from peak areas in the total ion chromatogram and represent mean values ​​obtained from a single GC–MS run per extract

The lipid profile observed was characterized by a predominance of SFAs with a significant contribution from MUFAs, particularly oleic acid, as well as the presence of PUFAs of biotechnological interest. The qualitative distribution shows a well-defined lipid pattern, with a predominance of C16 and C18.

### Thermal Analyses

#### Thermogravimetric Analyses (TGA)

**Fig. 1:**
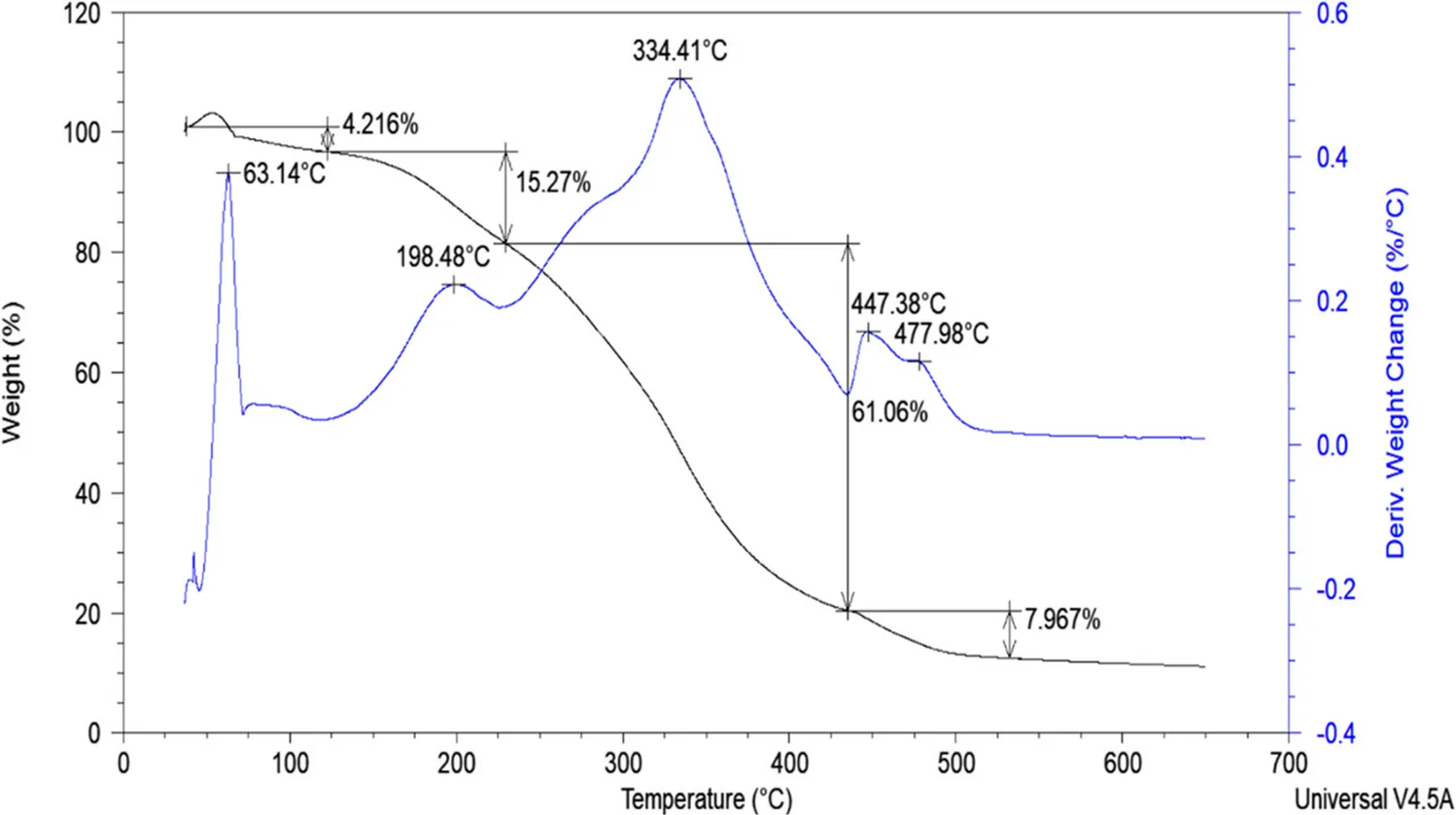
Thermogravimetric analysis curve of *Ulva ohnoi* lipid extract. The primary curve (black) illustrates weight loss (%) as a function of temperature; the derivative thermogravimetric curve (DTG, blue) shows the rate of weight change. Four thermal decomposition events were identified: Td₁ = 198 °C, Td₂ = 334 °C, Td₃ = 447 °C, and Td₄ ~ 477 °C. An initial volatile loss of ~ 4% was observed prior to Td₁, with a cumulative mass loss of approximately 88% at 650 °C. A single representative analysis is presented

The TGA results for *U. ohnoi* LE are shown in Fig. 1 and the corresponding thermal parameters are presented in Table 2. An initial mass loss of approximately 4% was observed in the *U. ohnoi* LE, which was attributed to the evaporation of volatile components, such as residual solvents and/or water. The thermal decomposition of the material occurred in a complex manner, evidencing four distinct degradation events. These events occurred at 198 °C (Td₁), 334 °C (Td₂), 447 °C (Td₃), and 477 °C (Td₄), resulting in a cumulative mass loss of approximately 88%.

**Table 2:** Thermogravimetric analysis (TGA) results for the lipid extract of *Ulva ohnoi* parameters of the lipid extract from *Ulva ohnoi*, including volatile mass loss and degradation temperatures at four thermal events

Lipid extract	Volatile (%) mass loss	T_d1_ (°C)	T_d2_ (°C)	T_d3_ (°C)	T_d4_ (°C)	Mass loss (%)
*Ulva ohnoi*	4	198	334	447	477	88%

Td₁, Td₂, Td₃, Td₄: temperatures (°C) at which each of the four main thermal degradation events occurs, as identified from the derivative thermogravimetric (DTG) curve. Volatile: initial mass loss (%) attributed to evaporation of residual solvents and/or water prior to thermal decomposition. Total mass loss (%): cumulative mass loss at 650 °C. A single representative run is shown

#### Differential Scanning Calorimetry (DSC)

**Fig. 2:**
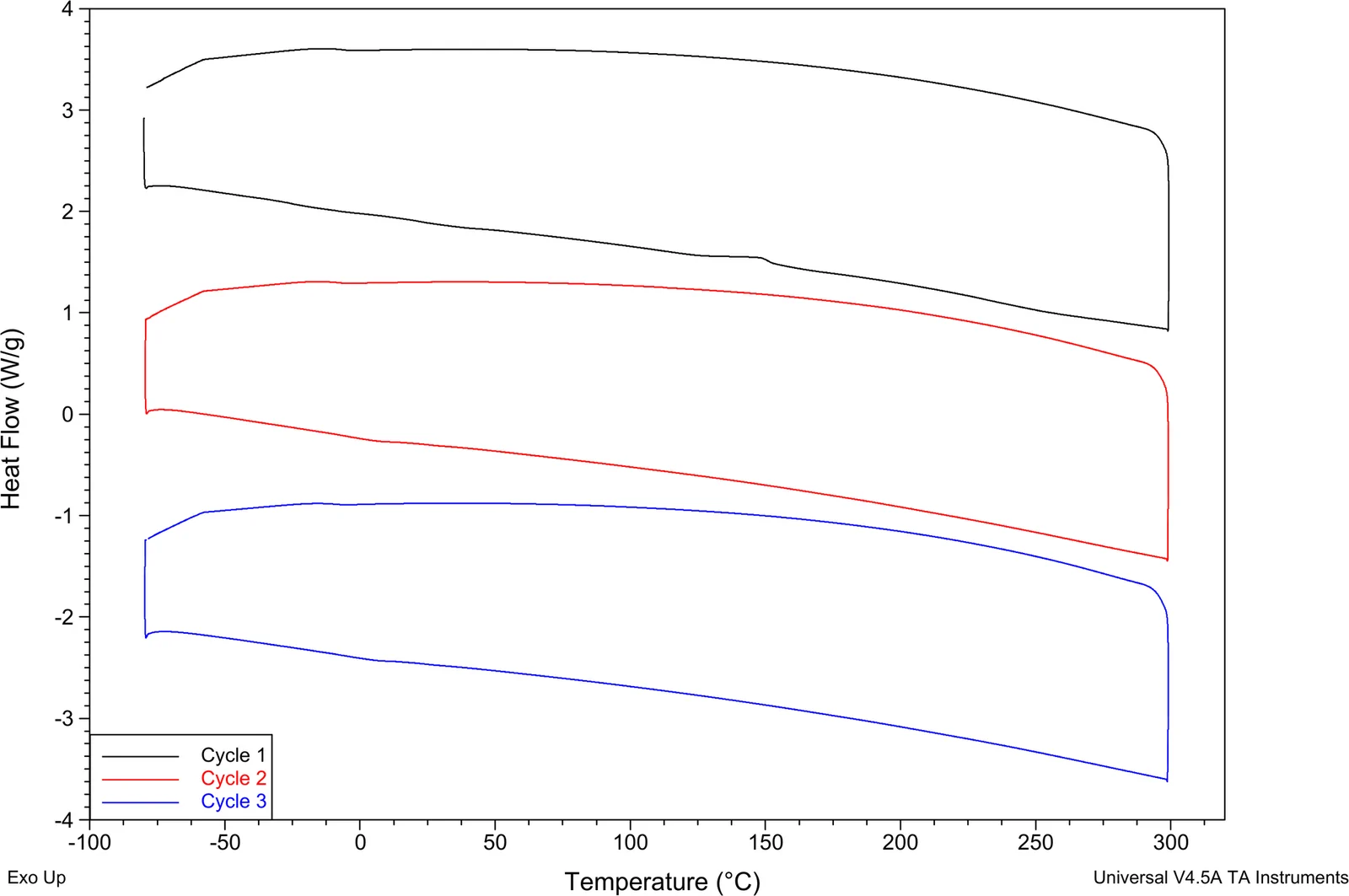
Modulated temperature differential scanning calorimetry (MTDSC) curve of *Ulva ohnoi* lipid extract. Three consecutive heating and cooling cycles are shown (Cycle 1: black; Cycle 2: red; Cycle 3: blue). The absence of endothermic or exothermic peaks across all cycles indicates no detectable thermal transitions (glass transition, crystallization, or melting) within the evaluated temperature range (− 80 °C to 300 °C), consistent with an amorphous lipid system without measurable crystalline domains. As with the TGA analysis, curves represent a single representative extract batch; error bars and statistical comparisons are not applicable to this curve-based thermal analysis

The *U. ohnoi* LE showed no significant thermal transitions when subjected to DSC over the temperature range of − 80 to 300 °C (Fig. 2). This finding indicates the absence of processes such as glass transition, crystallization, or melting, thereby suggesting that measurable amorphous or crystalline domains were not present in the extract under the conditions tested.

### Susceptibility of MDR Hospital Isolates

Table 3 summarizes the clinical resistance profiles of the MDR isolates used in this study, as determined by automated susceptibility testing (VITEK 2 and BD PHOENIX). These profiles confirm the MDR classification of all isolates with resistance to ≥ 3 antibiotic classes among the 16 antibiotic classes evaluated. Further details regarding the individual resistance profiles for each bacterial isolate are provided in Supplementary Table 1. The experimental antibacterial activity of *U. ohnoi* LE against these same isolates is presented separately in Table 4.

**Table 3:** Summary of MDR classification of hospital isolates: number of antibiotic classes with identified resistance (of 16 tested) and MDR status (resistance to ≥ 3 classes)

Species (n)	Gram	Antibiotic classes with resistance identified	No. of classes resistant (of 16 tested)
*Acinetobacter baumannii* (3)	GNB	Aminoglycosides β-lactams Inhibitors Carbapenems Cephalosporins Nitrofurans Penicillins Quinolones	≥ 6
*Escherichia coli* (3)	GNB	Aminoglycosides β-lactams Inhibitors Carbapenems Cephalosporins Penicillins Quinolones	≥ 4
*Klebsiella pneumoniae* (3)	GNB	Aminoglycosides β-lactams Inhibitors Carbapenems Cephalosporins Nitrofurans Penicillins Quinolones	≥ 5
*Pseudomonas aeruginosa* (3)	GNB	Aminoglycosides β-lactams Inhibitors Carbapenems Cephalosporins	≥ 3
*Staphylococcus aureus* (3)	GPC	Macrolides Lincosamides Penicillins Quinolones Rifampicin	≥ 3
Coagulase-negative staphylococci (3)	GPC	Aminoglycosides Glycopeptides Lincosamides Macrolides Penicillins Quinolones Rifampicin	≥ 5

Summary of MDR classification of hospital isolates: number of antibiotic classes with identified resistance (of 16 antimicrobial classes tested) and MDR status, defined as resistance to ≥ 3 antibiotic classes (Magiorakos et al. 2012). Three independent clinical isolates per bacterial species were evaluated (*n* = 3 isolates per species; 18 isolates in total). Detailed individual resistance profiles for each isolate are provided in Supplementary Table 1 (Supplementary Material). GNB: Gram-negative bacilli; GPC: Gram-positive cocci

### Antibacterial Activity

MIC and MBC values for each bacterial species are presented in Table 4. The MBC assay showed bacteriostatic action at the same concentrations identified in the MIC that were evaluated in the LE concentrations of *U. ohnoi*. Bacterial growth persisted in the subcultures at all tested concentrations for all isolates, up to the highest concentration evaluated (8 mg/mL); according to the criteria described in Materials and Methods, the MBC values ​​are therefore presented in Table 4 as exceeding that concentration (> 8.00 mg/mL).

**Table 4:** MIC and MBC of *U. ohnoi* lipid extract against MDR hospital-acquired isolates (resistance to ≥ 3 antibiotic classes; Magiorakos et al. 2012), with individual replicate values, mean ± SD, and predominant antibacterial action

Bacterial species (n)		Repetitions					
	Technical replicas	1ª	2ª	3ª	MIC ± SD (mg/mL)	MBC (mg/mL)	Predominant action
*Acinetobacter baumannii* (I)	*a*	2.00	2.00	2.00	2.00	> 8.00	Bacteriostatic
	*b*	2.00	2.00	2.00			
*Acinetobacter baumannii* (II)	*a*	2.00	2.00	2.00	2.00	> 8.00	Bacteriostatic
	*b*	2.00	2.00	2.00			
*Acinetobacter baumannii* (III)	*a*	2.00	2.00	2.00	2.00	> 8.00	Bacteriostatic
	*b*	2.00	2.00	2.00			
*Escherichia coli* (I)	*a*	2.00	2.00	2.00	2.00	> 8.00	Bacteriostatic
	*b*	2.00	2.00	2.00			
*Escherichia coli* (II)	*a*	2.00	2.00	2.00	2.00	> 8.00	Bacteriostatic
	*b*	2.00	2.00	2.00			
*Escherichia coli* (III)	*a*	2.00	2.00	2.00	2.00	> 8.00	Bacteriostatic
	*b*	2.00	2.00	2.00			
*Klebsiella pneumoniae* (I)	*a*	4.00	4.00	4.00	4.00	> 8.00	Bacteriostatic
	*b*	4.00	4.00	4.00			
*Klebsiella pneumoniae* (II)	*a*	4.00	4.00	4.00	4.00	> 8.00	Bacteriostatic
	*b*	4.00	4.00	4.00			
*Klebsiella pneumoniae* (III)	*a*	4.00	4.00	4.00	4.00	> 8.00	Bacteriostatic
	*b*	4.00	4.00	4.00			
*Pseudomonas aeruginosa* (I)	*a*	4.00	4.00	4.00	4.00	> 8.00	Bacteriostatic
	*b*	4.00	4.00	4.00			
*Pseudomonas aeruginosa* (II)	*a*	4.00	8.00	8.00	6.67	> 8.00	Bacteriostatic
	*b*	4.00	8.00	8.00			
*Pseudomonas aeruginosa* (III)	*a*	4.00	4.00	4.00	4.00	> 8.00	Bacteriostatic
	*b*	4.00	4.00	4.00			
*Staphylococcus aureus* (I)	*a*	2.00	1.00	1.00	1.33	> 8.00	Bacteriostatic
	*b*	2.00	1.00	1.00			
*Staphylococcus aureus* (II)	*a*	2.00	1.00	1.00	1.33	> 8.00	Bacteriostatic
	*b*	2.00	1.00	1.00			
*Staphylococcus aureus* (III)	*a*	2.00	1.00	1.00	1.33	> 8.00	Bacteriostatic
	*b*	2.00	1.00	1.00			
Coagulase-negative staphylococci (I*)*	*a*	2.00	1.00	1.00	1.33	> 8.00	Bacteriostatic
	*b*	2.00	1.00	1.00			
Coagulase-negative staphylococci (II)	*a*	2.00	1.00	1.00	1.33	> 8.00	Bacteriostatic
	*b*	2.00	1.00	1.00			
Coagulase-negative staphylococci (III)	*a*	2.00	1.00	1.00	1.33	> 8.00	Bacteriostatic
	*b*	2.00	1.00	1.00			

Three distinct clinical isolates (I, II, III) were evaluated per bacterial species. Each isolate was tested in technical duplicate ('a' and 'b') on three independent days ('1ª', '2ª', '3ª'), yielding six determinations per isolate (*n* = 6) for MIC and MBC calculation. MBC values expressed as " > 8.00" indicate that bacterial growth was recovered on subculture at every concentration tested, up to the highest concentration of the dilution series (8.00 mg/mL); a true bactericidal endpoint was therefore not reached within the tested range, and the MBC/MIC ratio exceeded the conventional cut-off of 4 that defines bactericidal activity (Pankey and Sabath 2004; Wald-Dickler et al. 2018), consistent with the bacteriostatic action reported for all isolates

### Cell Viability Assay

**Fig. 3:**
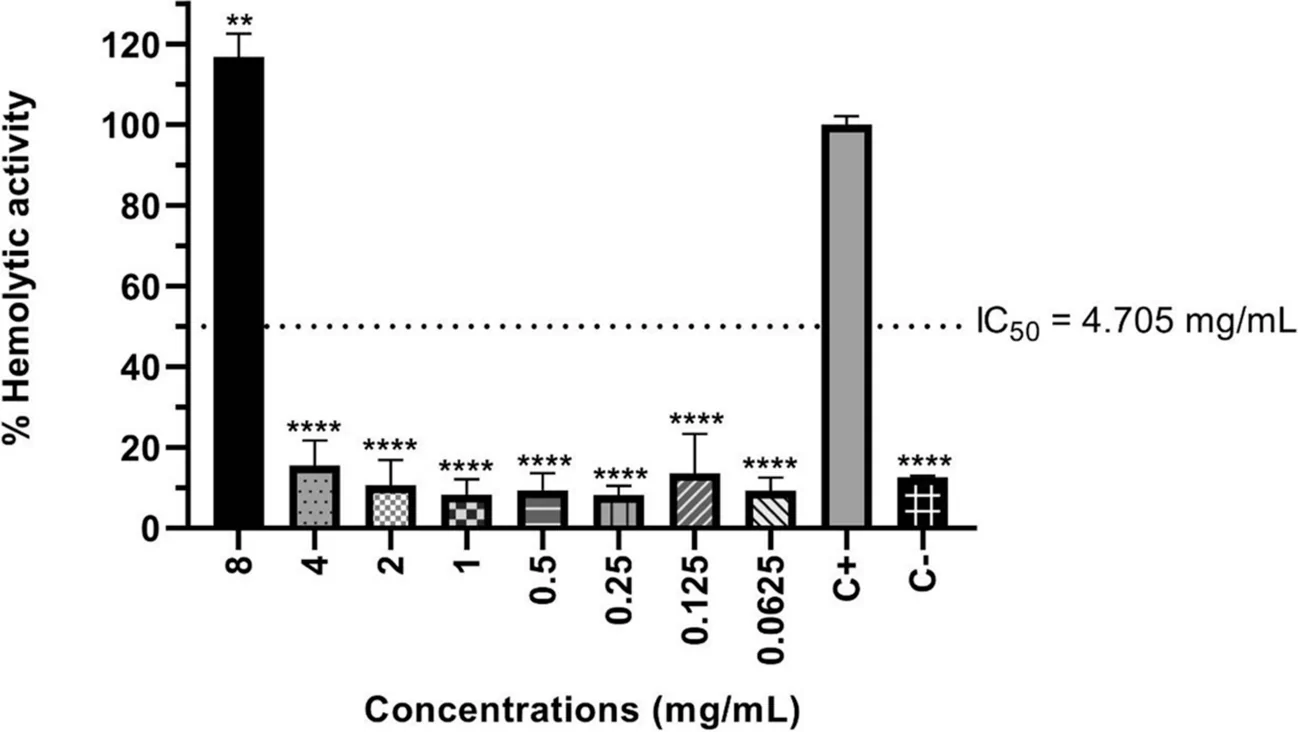
Hemolytic activity of lipid extract from *Ulva ohnoi*. Data are expressed as mean ± SEM (*n* = 3). Statistical comparisons were performed using two-way ANOVA followed by Sidak's post-hoc test. **p = 0.0043; ****p < 0.0001 compared to the positive control

To assess the safety of LE from *U. ohnoi* in systemic routes, its hemolytic potential against red blood cells was evaluated to estimate the selective toxicity of LE in blood cells. *U. ohnoi* LE demonstrated a promising safety profile in erythrocytes. As shown in Fig. 3, a significant increase in the percentage of hemolysis was observed only at the 8 mg/mL concentration. To confirm the absence of interference from the extract’s color at these concentrations, a photographic analysis of serial dilutions of the lipid extract (available in the Supplementary Fig. 1) demonstrated that the color did not cause any visible interference that would compromise the interpretation of the readings outside the 8 mg/mL concentration. Additionally, any potential influence of the color was mitigated by subtracting the absorbance of a blank sample, ensuring the reliability of the data obtained at the other concentrations. The IC_50_ value calculated for LE was 4.705 mg/mL (Table 5), reinforcing its low cytotoxicity against red blood cells and indicating a suitable profile for potential systemic applications.

**Table 5:** IC₅₀ values of *Ulva ohnoi* lipid extract from cytotoxicity assays (MTT, NR, and LDH) in MDBK and L929 cell lines and hemolysis assay (HA) in red blood cells, with overall mean and median IC₅₀ per cell line

Assays	Cell IC_50_​ (mg/mL)		Mean IC_50_ (mg/mL)	Median IC_50_ (mg/mL)	Cell IC_50_​ (mg/mL)		Mean IC_50_ (mg/mL)	Median IC_50_ (mg/mL)
	MDBK	Red blood cells			L929	Red blood cells		
HA		4.705	**1.8168**	**1.1552**		4.7050	**1.6338**	**0.79015**
MTT	0.3604				0.9984			
NR	1.9500				0.2499			
LDH	0.2520				0.5819			

Concentrations of 4 and 8 mg/mL were excluded from IC₅₀ calculation due to concentration-dependent optical interference from extract pigmentation in colorimetric assays (MTT and NR). MTT: [3-(4,5-dimethylthiazol-2-yl)−2,5-diphenyltetrazolium bromide] assay (mitochondrial activity); NR: Neutral Red uptake assay (lysosomal integrity); LDH: Lactate Dehydrogenase release assay (plasma membrane integrity); HA: Hemolytic Activity assay (erythrocyte membrane integrity); MDBK: bovine kidney cell line; L929: murine fibroblast cell line. Results are expressed as mean ± SEM

**Fig. 4:**
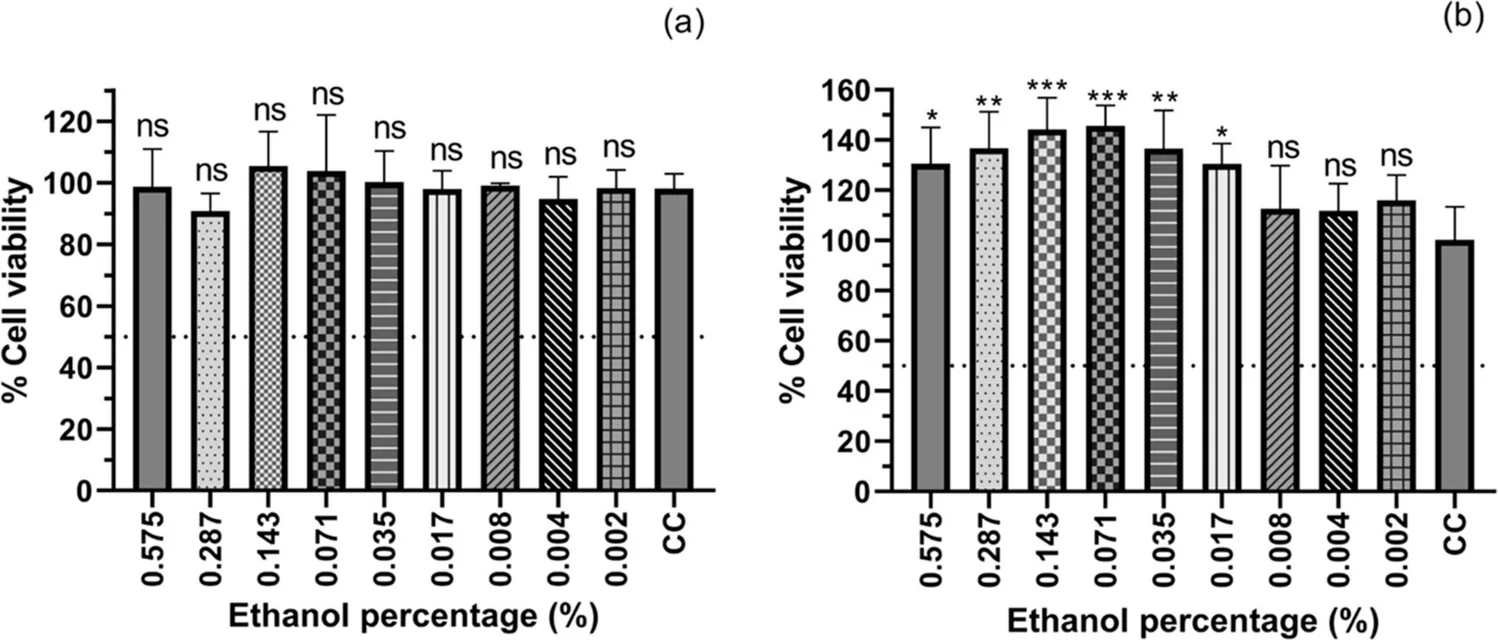
**a** MDBK Effect of ethanol (vehicle) on MDBK cell viability, assessed by the MTT assay. Data are expressed as mean ± SEM (*n* = 4). Two-way ANOVA followed by Sidak's post-hoc test. ns = not significant compared to the cell control (CC). **b **L929 Effect of ethanol (vehicle) on L929 cell viability, assessed by the MTT assay. Data are expressed as mean ± SEM (*n* = 4). Two-way ANOVA followed by Sidak's post-hoc test. *p = 0.0288; **p = 0.0054; ***p = 0.0003 (significant increases in viability at 0.575–0.017 mg/mL); ns = not significant compared to the cell control (CC)

The results of cell viability by MTT assay showed that exposure of MDBK and L929 cells (Fig. 4a and b) to decreasing concentrations of EtOH (0.575 to 0.002) did not cause a significant reduction in cell viability at any of the concentrations tested. In MDBK cells (Fig. 4a), viability remained close to 100% in all dilutions, with no significant difference from the control (untreated cells). In L929 cells (Fig. 4b), however, EtOH significantly increased cell viability relative to the control (approximately 130–145% of the control; *p* = 0.0003 to 0.0288) at concentrations from 0.575 to 0.017% (equivalent to LE concentrations of 2 to 0.0625 mg/mL), consistent with increased cell proliferation, whereas no significant difference was observed at 0.008, 0.004, and 0.002%. This indicates that EtOH, at the concentrations used to convey *U. ohnoi* LE, is not cytotoxic to the MDBK and L929 cell lines under the experimental conditions adopted, although at the higher concentrations it appeared to stimulate cell proliferation in L929 cells.

This indicates that EtOH, at the concentrations used to convey *U. ohnoi* LE, is not cytotoxic to the MDBK and L929 cell lines under the experimental conditions adopted. The results of the MTT assay showed that exposure of MDBK and L929 cells (Fig. 4a and b) was comparable to that of the control (untreated cells).

**Fig. 5:**
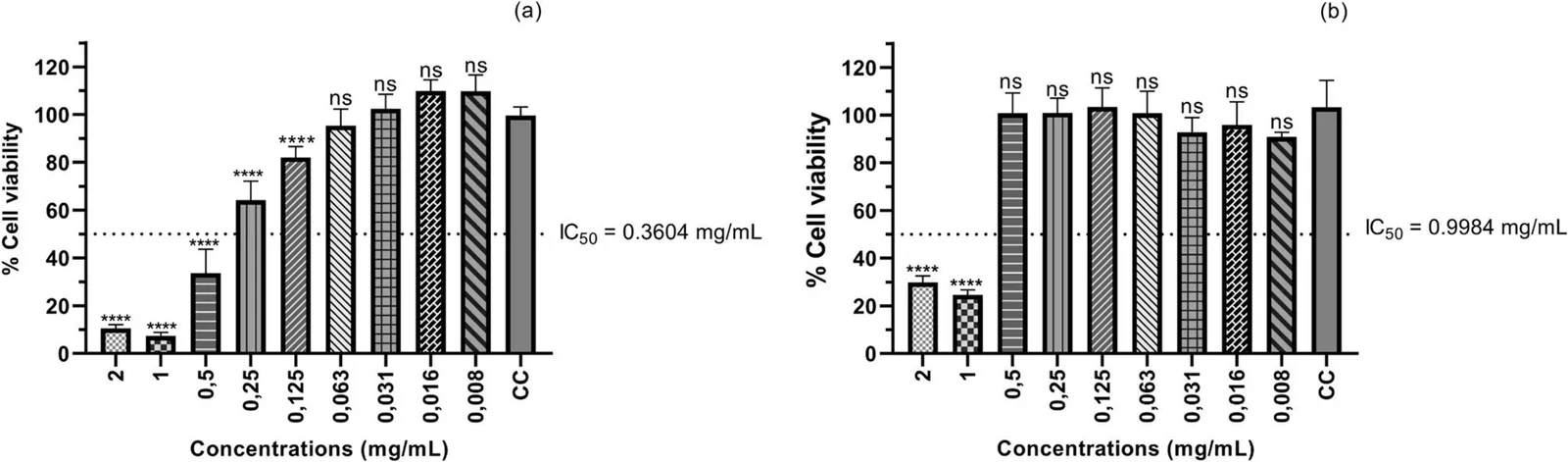
**a** MDBK cytotoxic activity of *Ulva ohnoi* lipid extract on MDBK cells, assessed by the MTT assay. Data are expressed as mean ± SEM (*n* = 4). Two-way ANOVA followed by Sidak's post-hoc test. ****p < 0.0001; ns = not significant compared to the cell control (CC). **b **L929 Cytotoxic activity of *Ulva ohnoi* lipid extract on L929 cells, assessed by the MTT assay. Data are expressed as mean ± SEM (*n* = 4). Two-way ANOVA followed by Sidak's post-hoc test. ****p < 0.0001; ns = not significant compared to the cell control (CC)

The MTT assay evaluates cell viability based on mitochondrial activity, and indicates the ability of cells to reduce the MTT mortality. *U. ohnoi* LE caused a significant reduction in MDBK cell viability (Fig. 5a) at concentrations ranging from 2.0 to 0.125 mg/mL, however, an increase in cell viability was observed from 0.063 to 0.008 mg/mL. In the L929 cell line (Fig. 5b), a significant reduction in cell viability was observed only at concentrations of 2 and 1 mg/mL. At lower concentrations, an increase in cell viability was observed between 0.5 and 0.008 mg/mL. Table 5 shows the estimated IC_50_ values, which were 0.3604 for MDBK and 0.9984 mg/mL for L929. The highest concentrations of 8 and 4 mg/mL were not included in the graphs (Fig. 5a and b) due to the probable interference of pigments present in LE, which impaired the measurement of optical absorbance. Similarly, the highest concentrations were also excluded from the graphical results for the NR and LDH methods. The morphological observations of the cells, performed by light microscopy, revealed changes consistent with the quantitative results obtained by the MTT and NR assays.

**Fig. 6:**
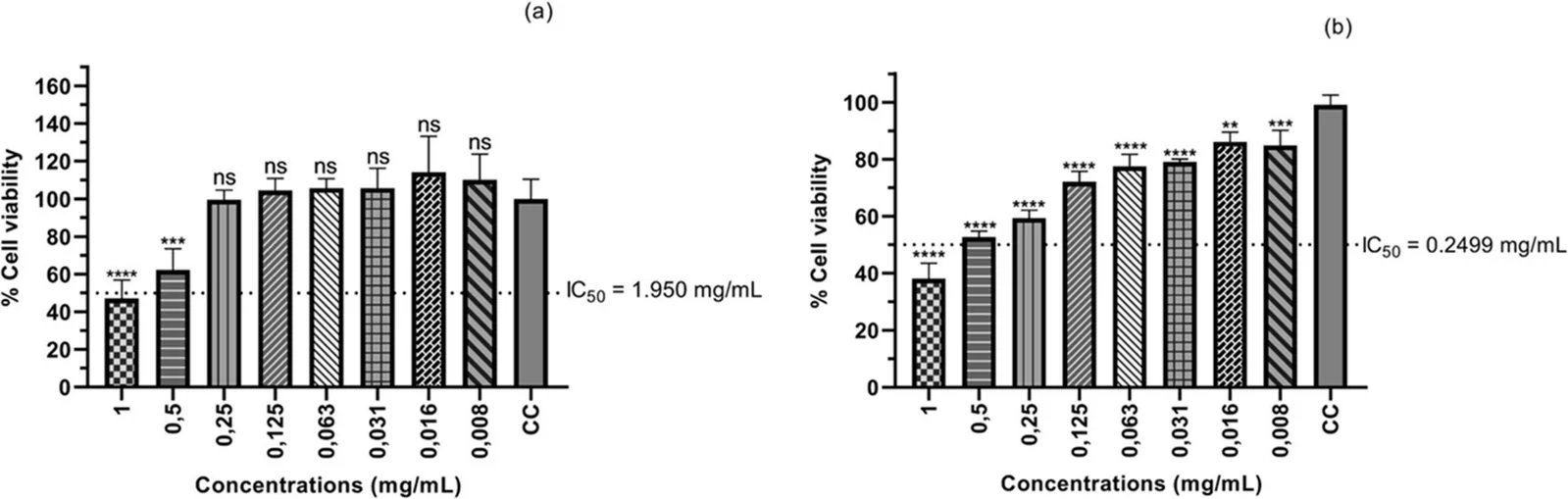
**a** MDBK Cytotoxic activity of *Ulva ohnoi* lipid extract on MDBK cells, assessed by the Neutral Red assay. Data are expressed as mean ± SEM (*n* = 4). Two-way ANOVA followed by Sidak's post-hoc test. ***p = 0.0003; ****p < 0.0001; ns = not significant compared to the cell control (CC). **b **L929 Cytotoxic activity of *Ulva ohnoi* lipid extract on L929 cells, assessed by the Neutral Red assay. Data are expressed as mean ± SEM (*n* = 4). Two-way ANOVA followed by Sidak's post-hoc test. **p < 0.0051; ***p < 0.0004; ****p < 0.0001 compared to the cell control (CC)

The NR method test which evaluates lysosomal integrity in MDBK cell lines against *U. ohnoi* LE showed a significant reduction in concentrations of 1.0 and 0.5 mg/mL. An increase and subsequent maintenance in cell viability from 0.25 to 0.08 mg/mL was observed, with no statistical difference from the cell control, as shown in the graphs in Fig. 6a. In contrast, L929 cells (Fig. 6b), shown a significant reduction in cell viability at all concentrations tested. The estimated IC_50_ values were 1.950 mg/mL for MDBK cells and 0.2499 mg/mL for the L929 cell line. MDBK cells were more resistant than L929 cells in the NR assay. The NR assay was effective in identifying changes in lysosomal integrity and was considered a reliable method for assessing cytotoxicity.

**Fig. 7:**
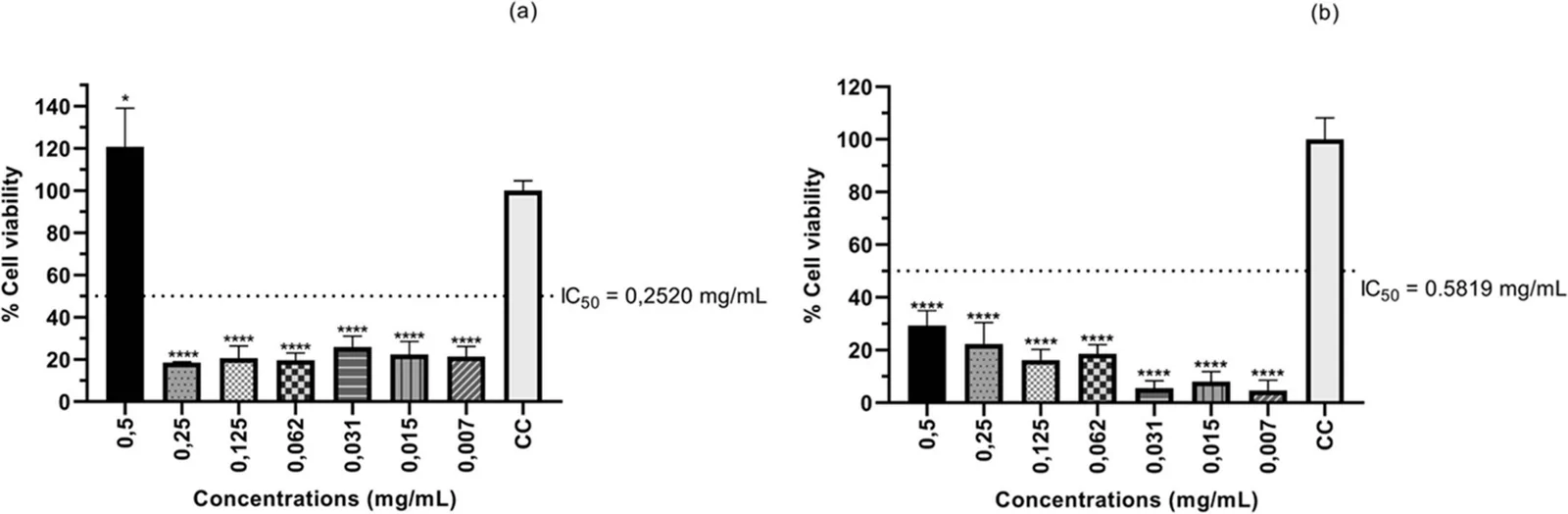
**a** MDBK cytotoxic activity of *Ulva ohnoi* extract on MDBK cells, assessed by the Lactate Dehydrogenase release assay. Data are expressed as mean ± SEM (*n* = 3). Two-way ANOVA followed by Sidak's post-hoc test. *p < 0.05; ****p < 0.0001 compared to the cell control (CC). **b **L929 cytotoxic activity of *Ulva ohnoi* lipid extract on L929 cells, assessed by the Lactate Dehydrogenase release assay. Data are expressed as mean ± SEM (*n* = 3). Two-way ANOVA followed by Sidak's post-hoc test. ****p < 0.0001 compared to the cell control (CC)

The LDH assay showed a significant reduction cell viability at concentrations ranging from 0.25 to 0.007 mg/mL, whereas no significant reduction was observed at 0.5 mg/mL. In L929 cells, a significant reduction was observed at all concentrations ranging from 0.5 to 0.007 mg/mL (Fig. 7a and b). Table 5 shows the IC_50_ values of 0.2520 mg/mL for MDBK cells and 0.5819 mg/mL for L929 cells. The rupture of the MDBK and L929 cell membranes as verified by the LDH supernatant assay using the MTT assay showed greater resistance of the L929 strain compared to MDBK when treated with *U. ohnoi* LE.

Table 5 presents the overall IC_50_ determined from cytotoxicity and HA assays. The mean and median IC_50_ values for cell viability were also calculated. L929 cells were more resistant than MDBK cells in the MTT and LDH assays, whereas MDBK cells showed greater resistance in the NR assay and also had higher mean and median IC_50_ values than those calculated for L929 cells across all assays. Thus, it was concluded that IC_50_ values varied according to the type of assay and cells, ranging from 0.2499 to 4.705 mg/mL. In addition, the HA assay showed that the LE of *U. ohnoi* did not cause significant hemolysis at the concentrations evaluated. Furthermore, L929 cells were generally more resistant to LE than MDBK cells.

The SI assessment, which relates cytotoxicity (IC_50_) to antibacterial activity (MIC), revealed that SI values for all bacterial strains tested were below 10, which is the established parameter for a safe therapeutic margin (Table 6). In fact, SI values ranged from 0.41 (for *K. pneumoniae* and *P. aeruginosa* in L929 cells) to 1.37 (for *S. aureus* and CoNS in MDBK cells). This indicates that the LE concentrations of *U. ohnoi* required to exhibit antimicrobial activity are close to or, in many cases, higher than the concentrations that cause cytotoxicity in mammalian cells. The greater resistance observed in L929 cells compared to MDBK cells for some bacterial species, as well as the low hemolysis observed in additional assays, suggest the possibility of a more favorable therapeutic window under specific conditions or through strategies involving delivery and chemical modification of the extract.

**Table 6:** Selectivity Index (SI = IC₅₀/MIC) of *Ulva ohnoi* lipid extract for each bacterial species, based on the mean IC₅₀ across cytotoxicity assays (MTT, NR, and LDH) and the mean MIC against MDR hospital isolates

Bacterial species	Mean MIC (mg/mL)	Mean cell MDBK IC_50_​ (mg/mL)	Mean cell L929 IC_50_​ (mg/mL)	SI cell MDBK	SI cell L929	Therapeutic margin
*Acinetobacter baumannii*	2.00	**1.82**	**1.63**	0.91	0.82	**SI < 10**
*Escherichia coli*	2.00			0.91	1.80	**SI < 10**
*Klebsiella pneumoniae*	4.00			0.45	0.41	**SI < 10**
*Pseudomonas aeruginosa*	4.00			0.45	0.41	**SI < 10**
*Staphylococcus aureus*	1.33			1.37	1.20	**SI < 10**
Coagulase-negative staphylococci	1.33			1.37	1.20	**SI < 10**

Mean IC₅₀ values were calculated combining the MTT, NR, and LDH assays for each cell line (MDBK and L929). SI ≥ 10 indicates an acceptable pharmacological safety margin; values below this threshold indicate an insufficient therapeutic margin. All SI values were below the threshold for every bacterial species tested. SI: Selectivity Index; MIC: Minimum Inhibitory Concentration; IC₅₀: half-maximal inhibitory concentration; MDBK: bovine kidney cell line; L929: murine fibroblast cell line

## Discussion

The LE of *U. ohnoi* was characterized by a predominance of SFAs, with palmitic acid accounting for 63.62% of the total FA content, whereas MUFAs and PUFAs were present in lower proportions. This FA may have contributed to the biological effects observed in the present study. Previous studies have shown that palmitic acid can induce cytotoxicity in different eukaryotic cells by promoting oxidative stress and activating apoptotic pathways, among others. In contrast, unsaturated FAs, especially oleic acid, have been reported to neutralize or attenuate the toxic effects of palmitic acid (Gómez-Lechón et al. 2007; Moravcová et al. 2015; Urso and Zhou 2021). Therefore, the ratio of palmitic acid to oleic acid observed in this study may have contributed to partially mitigating the cytotoxic effects associated with palmitic acid However, this possibility was not experimentally investigated and remains speculative.

TGA analysis revealed a multistep thermal decomposition profile for the LE of *U. ohnoi*, characterized by successive mass-loss events. The initial weight reduction was attributed to the release of volatile constituents, such as residual moisture, provides relevant information about the nature and degree of purity of the analyzed sample. Further increases in temperature led to additional degradation stages indicating the presence of different organic fractions in the LE, each with specific thermal stability and a characteristic decomposition temperature. This multi-stage degradation pattern reflects the chemical complexity of the material, whose cumulative mass loss at the end of the process highlights the extensive decomposition of the organic components present. Regarding phase transitions, DSC analysis did not detect significant thermal events within the investigated temperature range. The absence of endothermic or exothermic peaks associated with melting, crystallization, or glass transition suggests that the LE of *U. ohnoi* behaves predominantly as an amorphous system without the formation of structured domains on a measurable scale. This finding indicates that, under the experimental conditions adopted, the material does not exhibit crystalline organization or detectable phase separation, which is consistent with the behavior of complex lipid mixtures, in which interactions among components may suppress well-defined thermal transitions (Teles dos Santos et al. 2012; Tamási and Marossy 2022).

The thermal decomposition characteristics, mass-loss profiles, and absence of detectable phase transitions observed in this study provide, to the best of our knowledge, the first thermal characterization of LE from *U. ohnoi*. To date, no reports have been found in the literature on the thermal characterization by TGA and DSC of LE from algae of the genus *Ulva*, especially the species *U. ohnoi*. Most available studies focus on polysaccharide extracts or microalgal oils for biotechnological purposes. Therefore, the thermal parameters described here, including the decomposition stages, the characteristics of mass loss, and the absence of observed phase transitions, constitute a set of previously unreported data that significantly contributes to a better understanding of thermal stability and physicochemical properties of these extracts. This information may be useful for future biotechnological applications aiding in the definition of temperature limits for processing and storage, as well as providing insights into the behavior of these materials in pharmaceutical or cosmetic formulations.

From a drug-development perspective, thermal characterization is not merely descriptive. The multistep TGA degradation profile also indirectly relevant information to the thermal and oxidative stability of the extract, selecting compatible sterilization methods and predicting formulation shelf-life. Likewise, the amorphous behavior indicated by DSC is known to influence excipient compatibility and encapsulation strategy (e.g., liposomes, nanoemulsions) in lipid-based drug delivery systems (Chiu and Prenner 2011). Thus, thermal profiling is considered a standard preliminary step in the early-stage characterization of natural lipid-based antimicrobial candidates, preceding formulation and stability studies.

Free FAs, including palmitic acid and lauric acid, can exhibit antibacterial activity through multiple mechanisms, such as disrupting bacterial membrane integrity, interfering with membrane-associated processes, and altering membrane fluidity (Casillas-Vargas et al. 2021; Yoon et al. 2018). The absence of an outer membrane and the porous peptidoglycan layer of GPB may facilitate the interaction of these molecules with the bacterial membrane. In contrast, in GNBs, the presence of a thinner layer of peptidoglycan tied up with lipoproteins (proteins and lipopolysaccharides) and lipid A hinders the absorption of hydrophobic molecules (Barák and Muchová 2013; Epand and Epand 2009; Heesterbeek et al. 2019). This finding suggests that additional factors, including overall FA composition, relative abundance of individual lipids, and potential synergistic or antagonistic interactions among constituents, may also contribute to the antibacterial activity observed in this study. Furthermore, physicochemical properties such as chain length, degree of saturation, and double-bond configuration have previously been identified as factors affecting antimicrobial activity (Epand and Epand 2009; McGaw et al. 2002). These findings highlight the need for further studies to determine the specific contribution of the individual components present in the LE.

As the major constituent of the LE, and based strictly on prior literature rather than on mechanistic evidence generated in the present study, palmitic acid (C16:0) has been reported in previous studies to disrupt bacterial membrane integrity through its incorporation into lipid bilayers, increasing membrane rigidity and impairing proton-motive force (Desbois and Smith 2010; Yoon et al. 2018). However, high concentrations of palmitic acid are also associated with cytotoxicity in eukaryotic cells, a finding consistent with the low SI observed in this study. Oleic acid (C18:1n; 21.01%) has also been reported to exhibit antibacterial properties, particularly against Gram-positive organisms, through disruption of membrane fluidity (Petschow et al. 1996). It is important to emphasize that oleic acid has been shown to attenuate the cytotoxicity effects of palmitic acid in mammalian cells (Gómez-Lechón et al. 2007; Moravcová et al. 2015), as reflected in the palmitic:oleic ratio of approximately 3:1 observed here.

Although these findings provide plausible mechanistic explanations, the antibacterial activity evaluated in the present study was determined using the crude LE rather than isolated FAs. Therefore, the observed MIC values cannot be attributed to palmitic acid, oleic acid, or any other individual FA alone. The activity may result from synergistic or additive interactions between the full set of identified FAs, including minor PUFAs such as α-linolenic acid (C18:3) and stearidonic acid (C18:4), which have demonstrated antimicrobial properties in other contexts (Desbois et al. 2008). Further studies employing bioassay-guided fractionation and assays with isolated FA will be essential to establish definitive structure–activity relationships for this LE.

Considering that the antibacterial activity observed in the present study was evaluated from the crude LE, it is unlikely to be explained by the action of a single FAs. In addition to the major constituents, palmitic and oleic acids, other identified FAs, including palmitoleic, linoleic, α-linolenic, and stearidonic acids, have also been reported to exhibit antibacterial activity through membrane-targeting mechanisms. These compounds may therefore contribute to the overall bioactivity of the extract through additive or synergistic interactions (Desbois and Smith 2010; Casillas-Vargas et al. 2021).

Previous studies have shown that membrane disruption induced by bioactive lipids can increase bacterial permeability, facilitating the access of other bioactive constituents to intracellular targets and enhancing the overall antimicrobial effect. Nazzaro et al. (2013) reference other studies (Burt 2004; Chorianopoulos et al. 2008; Gutierrez et al. 2008; Marino et al. 2001) that corroborate these observations. As a result, bioactive compounds can penetrate damaged bacterial membranes and interfere with fundamental cellular processes, leading to the death or inactivation of bacterial cells (Berneira et al. 2021; Lopez-Romero et al. 2015).

In this context, the present study investigated the LE of *U. ohnoi*, a species whose lipid fraction remains relatively unexplored despite its reported antimicrobial properties. The three bacterial isolates evaluated for each species yielded similar results in both MIC and MBC assays and demonstrated bacteriostatic activity in all species and concentrations tested for antibacterial activity. The best MIC results exhibited bacteriostatic activity across all species and concentrations tested. The lowest MIC values were observed for *S. aureus* and CoNS (1.33 mg/mL), followed by *A. baumannii* and *E. coli* (2 mg/mL).

The activity observed for *U. ohnoi* LE should be interpreted in the context of a crude natural product extract. Because the preparation is a complex mixture, its MIC cannot be compared directly with the potency of purified antibiotics on a mass-for-mass basis. Clinical breakpoints established by CLSI/EUCAST (Clinical and Laboratory Standards Institute [CLSI] 2026; European Committee on Antimicrobial Susceptibility Testing 2026) were developed for defined antimicrobial agents and are not intended to establish direct clinical equivalence for hydrophobic, multicomponent extracts.

In natural product screening, a substantial proportion of the literature evaluates crude extracts in the mg/mL range and reserves µg/mL thresholds for purified compounds or later-stage fractions; review articles also emphasize that promising crude extracts should undergo bioactivity-guided fractionation, isolation of active constituents, and reassessment of potency and toxicity (Gonzalez-Pastor et al. 2023; Calefi et al. 2025).

Based on this, the MIC range reported in the present study is compatible with an early-stage, unfractionated extract and supports further fractionation rather than being interpreted as weak activity in the same sense used for purified ATBs (Gonzalez-Pastor et al. 2023; Calefi et al. 2025).

This pattern is supported by recent examples in the natural product literature: the crude ethanolic extract of *Calotropis procera* leaves showed MIC/IC_50_ values of 149–207 µg mL^−1^ against *S. aureus* and *E. coli*, whereas a purified enzyme (latex lysozyme) isolated from the same plant achieved MICs of only 13–14 µg mL^−1^ against a broader panel of pathogens — roughly a 10 to 15-fold potency gain from purification alone (Rodríguez-Macías et al. 2025). Nevertheless, even considering this context, the MIC values obtained (1.33–4.00 mg/mL) indicate moderate antibacterial activity, and a substantial improvement in potency through fractionation or purification of the crude LE will be required before any practical application can be considered.

To date, no studies have been identified evaluating the antimicrobial activity of *U. ohnoi* LE using the MIC method, however, only one study by Kang et al. (2021) that used *U. ohnoi* oil collected on an island in South Korea, showed antimicrobial activity conducted using the Ultrasensitive Radical Diffusion Assay method. Three oils (A, B, and C) were tested at a concentration of 10 mg/mL against *Bacillus subtilis* (KCTC1021), with a result of 10.36 mm for B and 7.35 mm for A, *E. coli* (isolate-D31) with a halo greater than 10 mm in all oil samples, and the fungus *Candida albicans* (KCTC76965) did not show halo formation.

In the present study, LE obtained from *U. ohnoi* biomass collected in Florianópolis-SC, Brazil, showed notable antibacterial activity against *E. coli*, with a MIC of 2 mg/mL. When compared with the antibacterial activity reported by Kang et al. (2021) against *E. coli* isolate-D31, the findings of the two studies, they complemented each other, as the oil from the experiment by Kang et al. (2021) and our LE from *U. ohnoi* presented positive antibacterial properties against the *E. coli* isolate. However, the antibacterial activity against *B. subtilis* and the antifungal activity against *C. albicans* reported by Kang et al. (2021) were not evaluated in the present study.

These findings should be interpreted in the context of a still limited body of literature on seaweed lipids. A recent systematic review screened 270 eligible publications on seaweed bioactive lipids and found that only 29 achieved a detailed molecular characterization of the compounds involved, underscoring how uncommon this level of chemical resolution remains in the field (Lopes et al. 2021). Within the genus *Ulva*, a relevant precedent is the study of *Ulva rigida* by Ismail et al. (2018), which bioguided fractionation identified oleic, linoleic, palmitic, and stearic acids as the main active constituents, with MIC values for the purified antibacterial fractions ranging from 10 to 250 µg/mL. Together, these studies support the view that the present work extends the field from general bioactivity screening toward chemical characterization and quantitative testing against clinically relevant MDR bacteria, complementing the findings of Kang et al. (2021) discussed above.

It should be emphasized that antibacterial activity in lipid fractions of *Ulva* species is not, in itself, a novel finding, as illustrated by the studies of Kang et al. (2021) and Ismail et al. (2018) discussed above. Therefore, the novelty of the present work lies in the comprehensive chemical characterization of *U. ohnoi* LE, together with its systematic evaluation against clinically relevant MDR isolates, rather than in the antibacterial property of *Ulva* lipids per se.

In the present study, IC₅₀ values were evaluated using four methods: LDH (membrane damage), MTT (metabolic activity), NR (lysosomal integrity), and HA (red blood cell toxicity) assays using two cell lines: The NR assay indicated greater sensitivity in L929 cells (IC_50_ = 0.2499 mg/mL) compared to MDBK cells (IC_50_ = 1.950 mg/mL). In contrast, the MTT assay showed more pronounced toxicity in L929 (IC_50_ = 0.9984 mg/mL) than in MDBK (IC_50_ = 0.3604 mg/mL), suggesting a possible difference in cellular metabolic response to LE.

The results of the MTT test with EtOH showed that the vehicle (EtOH at 15% v/v) used to dissolve the *U. ohnoi* LE was not cytotoxic to MDBK or L929 cells at the EtOH concentrations analyzed (0.575% to 0.002%; Fig. 4), as no significant reduction in viability was observed. In MDBK cells, EtOH did not differ significantly from the cell control at any concentration. In L929 cells, EtOH significantly increased cell viability at 0.575% to 0.017% (corresponding to LE concentrations of 2 to 0.0625 mg/mL), consistent with cell proliferation rather than a cytotoxic effect. Thus, the reductions in cell viability observed with the LE cannot be attributed to solvent cytotoxicity. This finding is relevant for the interpretation of the dose–response curves of LE, since the absence of EtOH cytotoxicity at these concentrations supports the reliability of the model and the conclusions about the biological activity of the extract. The LDH assay, which assesses plasma membrane damage, also showed distinct sensitivity between strains, with IC_50_ values of 0.5819 mg/mL (L929) and 0.2520 mg/mL (MDBK). The hemolysis assay, performed on red blood cells, showed a considerably higher IC_50_ value (4.705 mg/mL), suggesting good tolerability of blood cells with low hemolytic potential of *U. ohnoi* LE compared to its cytotoxicity in cultured cells. Although most studies on *Ulva* spp. have focused on conventional cell viability methods, the multidimensional approach adopted in the present study provides a more comprehensive assessment of the effects of *U. ohnoi* LE contributing to the development of protocols and potentially expanding its application in pharmacological and biotechnological areas, reinforcing the importance of detailed characterizations in macroalgae research.

The assessment of the SI, which is crucial for evaluating pharmacological safety, revealed that, for all bacterial strains tested, the values were below 10 (Table 6), the threshold established for a safe therapeutic margin. Specifically, SI values ranged from 0.41 (for *K. pneumoniae* and *P. aeruginosa* in L929 cells) to 1.37 (for *S. aureus* and CoNS species in MDBK cells). This indicates that the concentrations of the *U. ohnoi* LE required to exhibit antimicrobial activity are close to, or in many cases higher than, the concentrations that cause cytotoxicity in mammalian cells.

The greater resistance observed in L929 cells compared to MDBK cells for some bacterial species, as well as the low hemolysis observed in additional assays, suggest the possibility of a more favorable therapeutic window under specific conditions or through strategies involving administration and chemical modification of the extract.

The IC₅₀ results showed variable trends across the different assays (MTT, NR, LDH) and cell lines (L929 and MDBK), requiring a clear and integrated interpretation. This variation reflects that the extract may induce toxicity through distinct pathways, affecting metabolic activity (MTT), lysosomal integrity (NR), and plasma membrane permeability (LDH) to varying degrees and at different concentrations. For example, the higher sensitivity observed in a specific assay may indicate that the extract acts preferentially on a particular organelle or cellular process, while other aspects of the cell remain less affected.

Taken together, rather than being interpreted as isolated, assay-specific findings, the four endpoints provide a coherent, although partial, safety profile: The LDH and MTT assays indicate that plasma membrane integrity and mitochondrial/metabolic activity are the compartments most sensitive to the LE, the NR assay indicates comparatively later involvement of lysosomal function. The markedly higher IC_50_ obtained in the hemolysis assay indicates that erythrocyte membranes are comparatively less vulnerable than adherent cell membranes. This pattern is consistent with a lipid-driven mechanism whose effects may be related to cellular metabolic and membrane-remodeling activity and overall indicates that, while the LE does not show a selective, compartment-restricted mode of toxicity, its impact on nucleated, metabolically active cells is disproportionately greater than its impact on non-nucleated erythrocytes; this is the underlying reason why the favorable hemolysis result cannot, by itself, be taken to indicate a favorable overall SI.

The differences in sensitivity between the L929 (murine fibroblasts) and MDBK (bovine renal) cell lines (Table 5) are attributable to their physiological and biochemical characteristics. The differential resistance observed, where L929 proved to be more resistant in MTT and LDH assays, and MDBK in the NR assay, underscores the cell-type specificity of the cytotoxic response. These variations are expected, since different cells possess distinct detoxification capacities, receptor expression, and membrane composition, which may influence their interactions with the compounds present in the extract.

It is important to note that, in contrast to cytotoxicity in adherent cells, the hemolysis assay demonstrated a more favorable safety profile, with an IC₅₀ value of 4.705 mg/mL (Table 5), indicating good tolerability by blood cells. Although this finding suggests low hemolytic potential and a promising initial systemic safety profile, it is crucial to reconcile it with the low SI values derived from adherent cell assays. This discrepancy may suggest that the components of the extract have different modes of action or toxicity in suspended cells (erythrocytes) compared to adherent cells, or that mammalian adherent cells are inherently more sensitive to the cytotoxic effects of the LE compared to erythrocytes.

The dissociation between the results of the different assays observed in the present study is well documented in the toxicology literature; comparative studies using the same panel of cytotoxicity assays have similarly reported differential sensitivity among endpoints, with NR and MTT often detecting cytotoxic events at different concentration thresholds than LDH leakage (Fotakis and Timbrell 2006). Among the four assays performed in the present study, MTT was considered the most representative for a preliminary safety assessment, given its widespread use, validated mechanistic basis, and extensive precedent in the literature, and was therefore prioritized for calculation of the SI. Nonetheless, the lowest IC_50_ was observed in the LDH assay, while the most favorable safety margin was observed in the HA assay, reinforcing that a single assay is insufficient to characterize the safety profile of a crude, multi-component LE, and that the tiered, multi-assay, multi-cell-line approach adopted provides a more complete safety picture than any single test in isolation.

Additionally, the intense greenish staining of the LE, observed at higher concentrations (8 and 4 mg/mL), represented a methodological limitation in colorimetric assays such as MTT and NR. This pigmentation may have compromised the accuracy of the IC₅₀ determination in these ranges, due to the potential to mask optical readings and, consequently, affect the calculated IC₅₀ values. This reinforces the need for complementary approaches or purification of the extract for a more accurate assessment of its intrinsic cytotoxicity, as well as formulation strategies, such as encapsulation, to mitigate pigment interference and potentially increase the extract’s biological activity.

For each assay, curve fitting was based on a minimum of two concentrations above the estimated IC_50_ and several concentrations below it, all within the pigment-free readable range, which supported the reliability of the IC_50_ estimates despite the exclusion of the highest, pigment-affected concentrations. The inclusion of those higher concentrations would not be expected to shift the IC_50_ values, since the LE was already at or near maximal cytotoxicity at that point and the dose–response curves did not display a further exponential decline beyond it, indicating a toxicity plateau rather than the informative descending portion of the curve used for IC_50_ estimation. Nevertheless, IC₅₀ estimates obtained near the limit of the reliable concentration range should be interpreted with appropriate caution, and future studies using non-colorimetric, pigment-independent endpoints (e.g., flow cytometry-based viability assays) would allow more definitive quantification.

Given the moderate antibacterial potency and the currently low SI, systemic antimicrobial therapy is unlikely to be the most immediate practical application of the crude *U. ohnoi* LE. Other application routes that tolerate a narrower therapeutic window and rely primarily on local, rather than systemic activity may be more immediately relevant, including topical or wound-dressing formulations for localized infection control, incorporation into antimicrobial coatings for medical devices or surfaces, use as a natural preservative in antimicrobial food-packaging materials, and combined use with existing antibiotics to explore potential synergistic or adjuvant effects. These applications should be considered alongside, rather than as a substitute for, the fractionation and optimization strategies discussed above.

In summary, the multidimensional approach using multiple assays and cell lines allowed for a more comprehensive evaluation of the effects of *U. ohnoi* LE, revealing a complex safety profile that varies according to the assay and cell type. The discrepancies observed in IC₅₀ and IC₅₀ values, although challenging for direct interpretation, highlight the importance of considering multiple endpoints in cytotoxicity assessment to obtain a more comprehensive understanding of the effects of the extract on biological systems. Despite these complexities, the initial results regarding antimicrobial activity and the low hemolysis profile are promising, however, optimization of the extract and its formulation will be required to achieve a safer therapeutic margin.

## Conclusion

The LE from *U. ohnoi* demonstrated promising antimicrobial activity against all MDR strains tested, with the best results observed against *S. aureus* and CoNS.

Chemical characterization revealed that the LE was predominantly composed of SFAs, particularly palmitic acid, together with oleic acid and other FAs previously reported to exhibit antibacterial activity, providing a basis for future structure–activity investigations. In parallel, TGA revealed that the extract undergoes complex degradation in multiple events, indicating the presence of different organic fractions with distinct thermal stabilities. Additionally, DSC did not identify significant phase transitions, suggesting the absence of highly ordered crystalline domains under the experimental conditions evaluated.

Regarding safety, cytotoxicity assays (MTT, NR, and LDH) in MDBK and L929 cell lines showed IC₅₀ values of 0.25–1.95 mg/mL, whereas the HA assay indicated good tolerability of red blood cells (IC₅₀ = 4.705 mg/mL), with significant hemolysis only at the highest concentration tested (8 mg/mL). This favorable hemolysis result cannot, however, be taken as evidence of low systemic cytotoxicity, since the LE was cytotoxic to both mammalian cell lines at concentrations close to its MIC values. It is important to note that the ethanol vehicle control was not cytotoxic to either cell line and, in L929 cells, increased cell viability, consistent with cell proliferation, indicating that the reductions in viability observed with the LE cannot be attributed to the solvent.

However, the comprehensive pharmacological safety evaluation, including the SI, revealed values below the threshold for a safe therapeutic margin (SI ≥ 10), highlighting the need for further bioassay-guided fractionation, optimization and formulation strategies of the LE as a source of bioactive compounds. These findings reflect a preliminary, screening-level study, and the low SI values obtained should be interpreted as a starting point rather than a definitive indicator of therapeutic potential; further studies, including fractionation, structure–activity assessment, and formulation strategies to improve the therapeutic window, are needed before conclusions about clinical applicability can be drawn.

In addition, the intense greenish coloration of the LE at higher concentrations represented a significant methodological limitation in the colorimetric assays (MTT and NR), which may have affected the accuracy of the cytotoxicity measurements and the calculated SI values. In this regard, formulation strategies for the *U. ohnoi* LE may represent a useful approach to overcome pigment-related interference caused by pigments in the assays, as well as enhancing the extract’s biological activity; however, further studies are needed to ensure its efficacy and safety.

The antibacterial activity observed is more likely attributable to the combined action of multiple bioactive FA identified in the LE, including palmitic, oleic, palmitoleic, linoleic, α-linolenic and stearidonic acids, rather than to a single major constituent. Nevertheless, the relative contribution of each FA and the presence of additive or synergistic interactions within the crude extract remain unknown. Bioassay-guided fractionation and testing of isolated FA in future studies will be essential to establish definitive structure–activity relationships and to identify the active principle(s) responsible for the antimicrobial effects observed.

Furthermore, future investigations should include time-kill kinetics, biofilm inhibition assays, membrane permeability and depolarization assays, ultrastructural (SEM/TEM) analyses, resistance induction studies, and bioassay-guided fractionation, followed by purification of the individual active compounds, microtiter-plate synergy assays among the major FAs identified, and, ultimately, in vivo validation of efficacy and safety. These approaches would considerably strengthen the biological relevance of the findings and provide a more comprehensive understanding of the antibacterial mechanism and therapeutic potential of this promising marine-derived LE.

## Limitations

A methodological limitation identified in this study was the intense staining of the *U. ohnoi* LE, which directly interfered with the evaluation of cell viability, especially at the higher concentrations tested (8 and 4 mg/mL). This pigmentation made interpretation of the results from colorimetric techniques, such as MTT and NR, more difficult because of potential interference with optical readings. This interference could mask absorbance signals and therefore led to the exclusion of these concentrations from the presented graphs.

To mitigate this effect and ensure the reliability of the data at interpretable concentrations, experimental control strategies were employed. Specifically, cell-free controls were used to correct for background absorbance, allowing for the minimization of spectrophotometric interferences and a more precise quantitative analysis of cellular responses within the evaluated concentration ranges. Optical interference proved to be concentration-dependent, and was more pronounced at higher concentrations, whereas at subsequent concentrations below 4 mg/mL, the absorbance profile was compatible with the expected cellular response. Although we acknowledge the inherent limitations of colorimetric assays on highly pigmented extracts, the consistency of the observed results, the reproducibility among replicates, and the biological coherence of the dose–response curve, along with validation by multiple assays (MTT, NR, LDH), reinforce the interpretability of the data for the concentrations where the correction was effective. Future studies could benefit from purification of the extract or the use of alternative or complementary techniques that are less susceptible to pigment interference such as flow cytometry-based assays or encapsulation of LE in nanoparticles, to allow a more precise assessment of its intrinsic cytotoxicity.

A further limitation of the present study is the absence of time-kill kinetic data and serial passage assay results. The MIC and MBC endpoint methods employed provide validated measures of inhibitory and bactericidal concentrations but do not capture the temporal dynamics of bacterial growth inhibition nor assess the potential for resistance development following repeated exposure to sub-MIC concentrations of the extract. Time-kill curve analysis and serial passage experiments are planned as priority objectives in the continuation of this research and will be essential to characterize the mode and rate of bacteriostatic action and to evaluate the likelihood of resistance induction by *U. ohnoi* LE.

A significant methodological limitation of the MIC and MBC assays was the absence of reference antibiotics tested in parallel. The robust characterization of the MDR hospital isolates, obtained through automated susceptibility profiling across 16 antibiotic classes using VITEK 2 and BD PHOENIX, allowed the present study to focus on the intrinsic antibacterial activity of *U. ohnoi* LE. Nevertheless, the absence of conventional antimicrobial controls limits the possibility of directly comparing the efficacy of the LE with that of standard antibiotics under the same experimental conditions. Future studies should therefore include appropriate reference antibiotics as parallel controls, enabling a more precise assessment of the potency and antibacterial spectrum of *U. ohnoi* LE in relation to established antimicrobial treatments.

## Supplementary Information

Below is the link to the electronic supplementary material.

Supplementary file1 Photographic documentation of the color-interference control for the *Ulva ohnoi* lipid extract (LE) across the serial dilution range used in the cytotoxicity assays (8, 4, 2, 1, 0.5, 0.25, 0.125, and 0.0625 mg/mL) and the negative control (CN), tested in duplicate (A, B). Visible pigmentation of the extract (greenish-brown coloration) was evident only at the two highest concentrations tested (8 and 4 mg/mL); at concentrations ≤ 2 mg/mL, no visually detectable color interference was observed. This confirms that the extract's intrinsic pigmentation did not meaningfully affect the optical readings, and consequently the IC_50_ estimates, at the concentration range used for curve fitting (≤ 2 mg/mL) (PNG 860 KB)

Supplementary Table 1 Antibiotic resistance profiles of MDR hospital isolates (n = 3 per species) across 16 antimicrobial classes, as determined by automated susceptibility testing (VITEK 2 and BD PHOENIX) in two hospitals in Pelotas, Brazil, 2018–2019Antibiotic resistance profiles of MDR hospital clinical isolates (n = 3 independent isolates per bacterial species; 18 isolates in total) across 16 antimicrobial classes, as determined by automated susceptibility testing (VITEK 2, bioMérieux; BD Phoenix, Becton Dickinson) in two reference hospitals in Pelotas, Brazil (2018–2019). Isolates were classified as MDR based on resistance to ≥ 3 antibiotic classes (Magiorakos et al. 2012). R: resistant; S: susceptible; I: susceptible, increased exposure (intermediate); NT: not tested; NA: not applicable (class not represented in automated panel for this species). GNB: Gram-negative bacilli; GPC: Gram-positive cocci. These individual antibiotic susceptibility profiles are presented to document the specific basis on which each clinical isolate was classified as multidrug-resistant (resistance to ≥ 3 antibiotic classes), thereby justifying its inclusion among the MDR isolates selected for the antibacterial (MIC/MBC) assays against the U. ohnoi lipid extract; only isolates meeting this MDR criterion were used in the antimicrobial evaluation.

## Data Availability

The data that support the findings of this study are available from the corresponding author upon reasonable request.
