# Supplementary material for: Lipid Extract from Ulva ohnoi Algae: Chemical Characterization, Antibacterial Potential, and Cytotoxicity in the Search for New Agents Against MDR Infections
Source: Mar Biotechnol (NY). 2026 Oct 17;28(6):171. doi: 10.1007/s10126-026-10717-z (PMC13676642; doi:10.1007/s10126-026-10717-z)
Supplement: Supplementary file 1 [file 10126_2026_10717_MOESM2_ESM.docx]

***Marine Biotechnology***

**Lipid extract from *Ulva ohnoi* algae: chemical characterization, antibacterial potential, and cytotoxicity in the search for new agents against** **MDR infections**

Marisa Castro Jara¹,* (ORCID [0000-0002-9437-3558](https://orcid.org/0000-0002-9437-3558)), Diego Serrasol do Amaral² (ORCID 0000-0002-5436-2870), Leonardo Werner^2^ (ORCID 0009-0000-2027-6037), Eduardo Bastos^4^ (ORCID 0000-0001-6710-7281), Luana de Azevedo Aimi^4^ (ORCID 0009-0004-8682-7910), Nathalia Stark Pedra^6^ (ORCID 0000-0001-8662-7661), Vithor Parada Garcia^5^ (ORCID 0009-0005-1834-6370), Matheus Pereira de Albuquerque^5^ (ORCID 0009-0007-2175-2374), Clarissa Piccinin Frizzo^7^(ORCID 0000-0002-1175-7NT0), Milena de Nardin^7^ (ORCID 0009-0004-9NT8-54NA), Wellington da Rocha da Silva^3^ (ORCID 0000-0001-7460-1376), Silvia de Oliveira Hübner³ (ORCID 0000-0003-0482-95NA), Rodrigo de Almeida Vaucher^5^ (ORCID 0000-0003-3496-7335), Cláudio Martins Pereira de Pereira² (ORCID 0000-0002-2818-4281), Patrícia da Silva Nascente¹ (ORCID 0000-0002-3137-4107)

^¹^ Department of Microbiology and Parasitology, Institute of Biology, Federal University of Pelotas, Pelotas, RS, Brazil

^2^Department of Biochemistry and Bioprospecting, Federal University of Pelotas, Pelotas, RS, Brazil

³Department of Preventive Veterinary Medicine, Faculty of Veterinary Medicine, Federal University of Pelotas, Pelotas, RS, Brazil

^4^Department of Botany, Federal University of Santa Catarina, Florianópolis, SC, Brazil

^5^Laboratory of Research in Biochemistry and Molecular Biology of Microorganisms, Federal University of Pelotas, Pelotas, RS, Brazil

^6^Laboratory of Neurochemistry, Inflammation, and Cancer, Federal University of Pelotas, Pelotas, RS, Brazil

^7^Laboratory of Ionic Liquids and Nanomaterials, Department of Chemistry, Federal University of Santa Maria, Santa Maria, RS, Brazil

*****Correspondence to [marisa.jara@ufpel.edu.br](mailto:marisa.jara@ufpel.edu.br)

Department of Microbiology and Parasitology, Institute of Biology - Federal University of Pelotas (UFPel), Travessa André Dreyfus, n/n, Buildings 18 and 25, 96010-900, Capão do Leão, RS, Brazil

**Supplementary Table 1** Antibiotic resistance profiles of MDR hospital isolates (n = 3 per species) across 16 antimicrobial classes, as determined by automated susceptibility testing (VITEK 2 and BD PHOENIX) in two hospitals in Pelotas, Brazil, 2018–2019

| **Bacteria (n Isolates)** | **Classes of Antibiotics** | | | | | | | | | | | | | | | |
| --- | --- | --- | --- | --- | --- | --- | --- | --- | --- | --- | --- | --- | --- | --- | --- | --- |
|  | **Aminoglycosides** | **Β-Lactamase Inhibitors** | **Carbapenems** | **Cephalosporins** | **Glucopeptides** | **Glycylcyclines** | **Lincosamides** | **Lipopeptides** | **Macrolides** | **Nitrofurans** | **Oxazolidinones** | **Penicillins** | **Polypeptides** | **Quinolones** | **Rifampicines** | **Tetracyclines** |
| ***Acinetobacter baumannii* (I)** | S | R | R | R | NA | NA | NA | NA | NA | R | NA | R | S | R | NA | NA |
| ***Acinetobacter baumannii* (II)** | R | R | R | R | NA | S | NA | NA | NA | NA | NA | R | S | R | NA | NA |
| ***Acinetobacter baumannii* (III)** | R | R | S | R | NA | NA | NA | NA | NA | R | NA | R | NA | R | NA | NA |
| ***Escherichia coli* (I)** | S | R | R | R | NA | NA | NA | NA | NA | S | NA | R | NA | S | NA | NA |
| ***Escherichia coli* (II)** | R | R | S | R | NA | S | NA | NA | NA | NA | NA | R | S | R | NA | NA |
| ***Escherichia coli* (III)** | S | R | S | R | NA | NA | NA | NA | NA | S | NA | R | NA | R | NA | NA |
| ***Klebsiella pneumoniae* (I)** | S | R | R | R | NA | S | NA | NA | NA | NA | NA | R | NA | R | NA | NA |
| ***Klebsiella pneumoniae* (II)** | R | R | R | R | NA | NA | NA | NA | NA | R | NA | R | NA | R | NA | NA |
| ***Klebsiella pneumoniae* (III)** | R | R | R | R | NA | NA | NA | NA | NA | R | NA | R | NA | R | NA | NA |

(Continue)

**Supplementary Table 2** Continuation.

| **Bacteria (n Isolates)** | **Classes of Antibiotics** | | | | | | | | | | | | | | | |
| --- | --- | --- | --- | --- | --- | --- | --- | --- | --- | --- | --- | --- | --- | --- | --- | --- |
|  | **Aminoglycosides** | **Β-Lactamase Inhibitors** | **Carbapenems** | **Cephalosporins** | **Glucopeptides** | **Glycylcyclines** | **Lincosamides** | **Lipopeptídeos** | **Macrolides** | **Nitrofurans** | **Oxazolidinones** | **Penicillins** | **Polypeptides** | **Quinolones** | **Rifampicines** | **Tetracyclines** |
| ***Pseudomonas aeruginosa* (I)** | S | R | R | R | NA | NA | NA | NA | NA | NA | NA | NA | R | R | NA | NA |
| ***Pseudomonas aeruginosa* (II)** | S | S | S | R | NA | NA | NA | NA | NA | NA | NA | NA | R | R | NA | NA |
| ***Pseudomonas aeruginosa* (III)** | R | R | R | R | NA | NA | NA | NA | NA | NA | NA | NA | S | R | NA | NA |
| ***Staphylococcus aureus* (I)** | S | NA | S | S | S | S | R | S | R | NA | S | R | NA | NT | S | S |
| ***Staphylococcus aureus* (II)** | S | NA | NA | S | S | S | R | NT | R | NA | S | R | NA | R | R | NT |
| ***Staphylococcus aureus* (III)** | S | NA | NA | S | S | S | R | NT | R | NA | S | R | NA | R | R | NT |
| **Coagulase-negative Staphylococci (I)** | R | NA | NA | NT | R | S | R | S | NT | NA | S | R | NA | I | R | NT |
| **Coagulase-negative Staphylococci (II)** | NT | NA | NA | R | S | NT | R | NT | R | NA | NT | R | NA | R | R | NT |
| **Coagulase-negative Staphylococci (III)** | NT | NA | NA | R | S | NT | R | NT | R | NA | NT | R | NA | R | I | NT |

Antibiotic resistance profiles of MDR hospital clinical isolates (n = 3 independent isolates per bacterial species; 18 isolates in total) across 16 antimicrobial classes, as determined by automated susceptibility testing (VITEK 2, bioMérieux; BD Phoenix, Becton Dickinson) in two reference hospitals in Pelotas, Brazil (2018–2019). Isolates were classified as MDR based on resistance to ≥ 3 antibiotic classes (Magiorakos et al. 2012). R: resistant; S: susceptible; I: susceptible, increased exposure (intermediate); NT: not tested; NA: not applicable (class not represented in automated panel for this species). GNB: Gram-negative bacilli; GPC: Gram-positive cocci. These individual antibiotic susceptibility profiles are presented to document the specific basis on which each clinical isolate was classified as multidrug-resistant (resistance to ≥ 3 antibiotic classes), thereby justifying its inclusion among the MDR isolates selected for the antibacterial (MIC/MBC) assays against the *U. ohnoi* lipid extract; only isolates meeting this MDR criterion were used in the antimicrobial evaluation.
